# Basic Characterization of Natural Transformation in a Highly Transformable *Haemophilus parasuis* Strain SC1401

**DOI:** 10.3389/fcimb.2018.00032

**Published:** 2018-02-08

**Authors:** Ke Dai, Lvqin He, Yung-Fu Chang, Sanjie Cao, Qin Zhao, Xiaobo Huang, Rui Wu, Yong Huang, Qigui Yan, Xinfeng Han, Xiaoping Ma, Xintian Wen, Yiping Wen

**Affiliations:** ^1^Research Center of Swine Disease, College of Veterinary Medicine, Sichuan Agricultural University, Chengdu, China; ^2^College of Veterinary Medicine, Sichuan Agricultural University, Chengdu, China; ^3^Department of Population Medicine and Diagnostic Sciences, College of Veterinary Medicine, Cornell University, Ithaca, NY, United States; ^4^Sichuan Science-Observation Experimental Station of Veterinary Drugs and Veterinary Diagnostic Technology, Ministry of Agriculture, Chengdu, China

**Keywords:** *Haemophilus parasuis*, CRP, uptake signal sequence, natural competence, transformation

## Abstract

*Haemophilus parasuis* causes Glässer's disease and pneumonia, incurring serious economic losses in the porcine industry. In this study, natural competence was investigated in *H. parasuis*. We found competence genes in *H. parasuis* homologous to ones in *Haemophilus influenzae* and a high consensus battery of Sxy-dependent cyclic AMP (cAMP) receptor protein (CRP-S) regulons using bioinformatics. High rates of natural competence were found from the onset of stationary-phase growth condition to mid-stationary phase (OD_600_ from 0.29 to 1.735); this rapidly dropped off as cells reached mid-stationary phase (OD_600_ from 1.735 to 1.625). As a whole, bacteria cultured in liquid media were observed to have lower competence levels than those grown on solid media plates. We also revealed that natural transformation in this species is stable after 200 passages and is largely dependent on DNA concentration. Transformation competition experiments showed that heterogeneous DNA cannot outcompete intraspecific natural transformation, suggesting an endogenous uptake sequence or other molecular markers may be important in differentiating heterogeneous DNA. We performed qRT-PCR targeting multiple putative competence genes in an effort to compare bacteria pre-cultured in TSB++ vs. TSA++ and SC1401 vs. SH0165 to determine expression profiles of the homologs of competence-genes in *H. influenzae*. Taken together, this study is the first to investigate natural transformation in *H. parasuis* based on a highly naturally transformable strain SC1401.

## Introduction

*Haemophilus parasuis* (*H. parasuis*, HPS), a Gram-negative, non-spore-forming, pleomorphic bacterium of the *Pasteurellaceae* group. This bacterium is normally a benign swine commensal but may become a deadly pathogen upon penetration into multiple tissues, and causes Glässer's disease and pneumonia in pigs. Clinical signs of this illness are characterized by polyarthritis, fibrinous polyserositis, and meningitis, resulting in serious economic losses in the pork industry throughout the world (Oliveira and Pijoan, 2004; Huang et al., 2016; Zhang P. et al., 2016; Zhou et al., 2016). *H. parasuis* has been a major challenge for the worldwide pork industry (Zhao et al., 2013).

Horizontal gene transfer (HGT), or the process of swapping lateral genetic material between neighboring “contemporary” bacteria occurs through three main mechanisms: conjugation, transduction, and natural transformation (Johnsborg et al., 2007; Mell et al., 2011). Many bacteria are naturally competent (Johnston et al., 2014; Mell and Redfield, 2014). In natural transformation, exogenous fragments of DNA are taken up by a bacterial host from their immediate environment. Exogenous DNA markers may then subsequently become expressible (as re-circularized plasmids or recombined into chromosome) (Lorenz and Wackernagel, 1994). These exogenous DNA genes may change the recipient host's genotype and phenotype, or on the contrary, be degraded and utilized in DNA repair mechanisms triggered by antibiotic and UV radiation induced damage (Johnsborg et al., 2007; Dorer et al., 2011). Like *Haemophilus influenzae* and many other competent species, certain *H. parasuis* strains can actively take up environmental DNA under natural conditions, but not all isolates are competent. Besides, transformation frequencies vary widely from strain to strain as observed in *Actinobacillus pleuropneumoniae* (Bossé et al., 2009). Through a specific uptake mechanism, DNA fragments often escape degradation to integrate into the host genome by RecA-driven homologous recombination (Wu et al., 2015), thus allowing bacterial strains to acquire and express new genes (such as virulence factors and drug resistance genes). Using the ability of *H. parasuis* to take up DNA, we and others have developed gene knockout methods and measures of screening a variety of virulence factors (Bigas et al., 2005; Zhang et al., 2015; Li J. et al., 2016).

cAMP, as a second messenger, is required to facilitate natural DNA uptake in many species, such as in *Vibrio* and in *H. influenzae* (Wise et al., 1973; Macfadyen et al., 1998; Wu et al., 2015). cAMP and its receptor protein, CRP (cAMP receptor protein), as the major player in carbon-catabolite repression (CCR) in *H. influenzae*, can actively induce an array of competence genes, such as one of the DNA-uptake batteries: *comABCDE* operon. TfoX/Sxy (two homologs) play an important role as a transcription co-regulator for localization of cAMP-CRP complexes to competence-dedicated CRP-S sites (a 22-bp sequence in several competence-related genes. See results below), thus increasing DNA uptake (see a recent review by Seitz and Johnston) (Seitz and Blokesch, 2013; Johnston et al., 2014). However, in *H. parasuis*, the underlying molecular mechanisms of DNA uptake/transformation have not been well-studied. To further understand the new insights into natural competence mechanisms in *H. parasuis*, we focus on bioinformatics analysis in the search for the latent competence-related genes and their potential involvement in regulating natural competence in this species.

## Materials and methods

### Strains, plasmids, and growth conditions

The bacterial strains and plasmids used in this study are listed in Table 1. Plasmids were propagated in *Escherichia coli* DH5α and the bacteria were grown in liquid Luria-Bertani (LB, Difco, USA) medium or on LB agar (Invitrogen, China) plate supplemented with optimal adaptive antibiotic concentrations (kanamycin, 50 μg ml^−1^ or ampicillin, 100 μg ml^−1^ from Sigma-Aldrich, USA) when required. *H. parasuis* was grown in Tryptic Soy Broth (TSB, Difco, USA) or on Tryptic Soy agar (TSA, Difco, USA) supplemented with 5% inactivated bovine serum (Solarbio, China) and 0.1% (*w/v*) nicotinamide adenine dinucleotide (NAD, Sigma-Aldrich, USA) (TSB++ and TSA++). Where necessary, the media were supplemented with 50 μg ml^−1^ of kanamycin. All strains were grown at 37°C. The serotype of SC1401 was identified using PCR based molecular serotyping (Howell et al., 2015), supplemented with agar gel diffusion precipitation (AGP) for further confirmation of the serotype.

**Table 1:** Bacterial strains and plasmids used in this study.

**Strain or plasmid**	**Relevant characteristic(s)**	**Source or reference**
**STRAINS**		
***H. parasuis***		
SC1401	Serotype 11 clinical isolate (Sichuan, China), highly transformable strain	Dai et al., 2016
EP3	Serotype 13 clinical isolate (Sichuan, China), transformable strain	He et al., 2016
SH0165	Serotype 5 clinical isolate (Hebei, China), non-transformable strain	Yue et al., 2009
SC1401Δ*htrA*::kan	SC1401 derivative, *hphtrA* deletion, kanamycin resistant	Zhang L. et al., 2016
***A. pleuropneumoniae***		
SC1516	Serotype 7 clinical isolate	Li Y. et al., 2016
***H. influenzae***		
QHH	Clinic isolate (Shanghai, China)	Laboratory collection
***Bacillus subtilis***		
WR	Clinic isolate (Sichuan, China)	Laboratory collection
***S. pneumoniae***		
WZH	Clinic isolate (Sichuan, China)	Laboratory collection
*E. coli* ZY16	Clinical isolate, enterotoxigenic *E. coli*	Laboratory collection
DH5α	FΦ80ΔlacZΔM15Δ(lacZYA-argF) U169 recA1 endA1 hsdR17	Laboratory collection
**PLASMIDS**		
pMD-19T(simple)	Amp^R^, T-vector	Takara
pK18mobsacB	Kan^R^; suicide and narrow-broad-host vector	Laboratory collection
pMDHK	Amp^R^, Kan^R^; a 2153-bp fragment containing the motif of 5′-ACCGCTTGT and the Δ*htrA*::kan cassette in pMD19-T	Zhang et al., 2015; Zhang L. et al., 2016
pKBHK	Kan^R^, a 2153-bp fragment containing motif of 5′-ACCGCTTGT and the Δ*htrA*::kan cassette in pK18mobsacB	Zhang et al., 2015

### Natural transformation methodology

The protocol for natural transformation was performed according to Bigas with some modifications. Briefly, recipient bacteria were grown in TSB++ to an optical density 600 (OD_600_) = 1.8 (about 2.9 × 10^9^ cfu/mL), a late-stationary phases. The bacteria were then spotted on TSA++, cultured overnight at 37°C and resuspended in TSB++ at ~2 × 10^10^ cfu/mL. A 20 μL aliquot of suspension was supplemented with 1 μL of cAMP (final concentration of 8 mM) and 2 μg of donor DNA (which has a maximum transformation efficiency in *H. parasuis* SC1401, data not shown). The mixture was incubated for 10 min at 37°C and then spotted on TSA++ again, for 5-h incubation at 37°C to induce expression of antibiotic resistance. Afterwards, bacteria were harvested and resuspended in 100 μL of TSB++ and plated on selective medium after serial dilution. The diluted bacteria (about 10^−8^) were plated on TSA++. Bacteria were incubated for 1–2 days. Colonies were identified by PCR; the RNA of the isolates was further identified using RT-PCR.

### *In vitro* growth characteristic of SC1401

To study the *in vitro* growth kinetics of wild-type *H. parasuis* SC1401, overnight-grown seed broth was subcultured 1:100 in fresh TSB++ at 37°C under aerobic or anaerobic conditions. Their OD_600_-values were measured at 1-h interval. Viable colonies of different OD_600_ were quantified manually after plating serial dilutions onto TSA++ plates.

### Stability of natural transformation after continuous passage

Wild-type SC1401 was cultured in 5 mL of TSB++ at 37°C with agitation for 12 h. The F2 (filial generation 2) was subcultured (1:100 dil.) in 5 mL of fresh TSB++ again. Thus, SC1401 was continuously passaged to 200 generations (F200). Natural transformation frequencies of every 10 generations were routinely monitored as above. Natural transformation analyses were performed in triplicate.

### Natural transformation in TSB++ and competence inducing phase analysis

We further studied whether natural competence can be induced in broth and determined competence rates calculated using natural transformation frequencies (TF, [kanamycin-resistant (Kan^r^) colony-forming units (CFU)/ total CFU]) of bacteria pre-cultured in broth. Briefly, recipient bacteria were grown in TSB++ to different growth phases monitored by OD_600_-values and colony forming units (from early logarithmic phase to the decline phase along the bacterial growth curve), harvested at 4,350 × g for 5 min, and resuspended in 20 μL TSB++ to keep the OD_600_ from 5 to 50. One microliter of cAMP (final concentration of 8 mM) and 2 μg of genomic DNA of SC1401Δ*htrA*::kan (Zhang L. et al., 2016) were added, the mixture was incubated for 30 min at 37°C; after that, the mixture was spotted on TSA++ again, incubated for 5 h at 37°C to induce expression of antibiotic resistance. The transformation frequency was calculated and evaluated as described below. All experiments were performed in triplicate.

### Transformation of *H. parasuis* SC1401 by chromosomal DNA from homologous and heterologous strains

The specificity of *H. parasuis* transformation by homologous DNA was assessed in competitive binding experiments as previously described with some modifications (Israel et al., 2000; Liu et al., 2017). Briefly, cells of wild-type strain SC1401 were incubated with a constant amount (1 μg) of SC1401Δ*htrA*::kan chromosomal DNA (with a Kan resistance marker) mixed with increasing amounts (0, 10, 50, 100, 500, 1,000, and 2,000 ng) of competitive DNA from either homologous or heterologous strains, including *H. parasuis* strains SC1401 and SH0165 (Li Y. et al., 2016), *A. pleuropneumoniae* strain SC1516, *E. coli* strain ZY16, *Bacillus subtilis* strain WR, and *Streptococcus pneumoniae* strain WZH. Host cells exposed to DNA from various sources were processed and plated on selective media; transformation frequencies of the DNAs were calculated.

### RNA preparation and RT (reverse transcription)-PCR

Molecular identification of transformants was performed using reverse transcription PCR (RT-PCR). Whole genomic DNA was extracted using TIANamp Bacteria DNA Kit (Tiangen, China). RNA was prepared using RNAprep pure Cell/Bacteria Kit (Tiangen, China). RT-PCR was performed using PrimeScript^TM^ RT-PCR Kit (TaKaRa, Japan). T4 DNA polymerase was purchased from Vazyme, China. Procedures were preformed following *Molecular Biology* and the manufacturer's protocols (Fu et al., 2016; Huang et al., 2016).

### Quantitative real-time PCR

RNA samples were isolated from the wild type SC1401 (cultured in TSB++ to obtain an OD_600_ = 1.46 and on TSA++ for 13 h, respectively), SH0165 (cultured on TSA++ plates for 13 h) using the RNAprep pure Cell/Bacteria Kit (Tiangen, China) according to the manufacturer's instructions. Two-step RT-PCR was performed using PrimeScript^TM^ RT reagent Kit with gDNA Eraser (Takara, Japan). The candidate natural transformation-related or dedicated gene transcripts were then analyzed using quantitative reverse transcription PCR (qRT-PCR) with the reagent of SYBR Premix EX Taq^TM^ II (Tli RNaseH Plus) (Takara, Japan). Primers for qPCR are listed in Table S1. Non-conserved primers with mismatched bases between SC1401 and SH0165 were designed separately. The 2(–ΔΔC(T)) method was used to relatively quantitate multiple genes expression compared to the stably expressed 16S RNA reference gene. qPCR runs were performed with a Lightcycler96 (Roche, Switzerland) system. The experiments were independently performed at least three times in triplicate.

### Statistical analysis

After incubation, the colonies on TSA++ and on selective plates were counted. Transformation frequencies were determined from the number of antibiotic-resistant cfu mL^−1^ divided by the total cfu mL^−1^ scored on non-selective agar (Israel et al., 2000; Humbert et al., 2011). Each experiment was independently performed at least three times in triplicate.

Comparisons of several test series were evaluated by analysis of variance (ANOVA tests). Multiple comparisons between any two means of different groups were performed using least significant difference (LSD) method. A *p* < 0.05 is considered to be statistically significant (^*^), and <0.01 highly significant (^**^), while the *p* > 0.1 is considered as statistically insignificant.

## Results

### Screening for isolates with detectable levels of natural transformation

Among the 30 clinical isolates and all 15 reference strains tested using the targeting vector pMDHK (Zhang et al., 2015), only five competent strains were identified (Table S2), indicating a different transformation pattern compared to *Neisseria gonorrhoeae* and *N. meningitidis* nearly all strains of which appear to be competent (Sparling, 1966; Mell and Redfield, 2014). This may be due to restriction barriers, differences in uptake sequences (USS, see below), diverse inducing cues, various evolutionary relationships and issues with replication in the case where plasmids are the transforming DNA (Solomon and Grossman, 1996; Johnsborg et al., 2007; Seitz and Blokesch, 2013; Mell and Redfield, 2014). Among these five transformable isolates, we found clinical strain SC1401 showed the highest frequency of natural competence (about 1.0 × 10^−4^) (Ding et al., 2016). We therefore sequenced the genome of SC1401 using Pacbio sequencer (Beijing Novogene Bioinformatics Technology Co., Ltd.) by single molecule real-time (SMRT) technology (GenBank accession NO: CP015099.1) (Dai et al., 2016). SC1401 was identified to be serotype 11 according to the PCR assay for molecular serotyping (Howell et al., 2015).

### SC1401 genome contains intact homologs of competence genes

**Figure 1:**
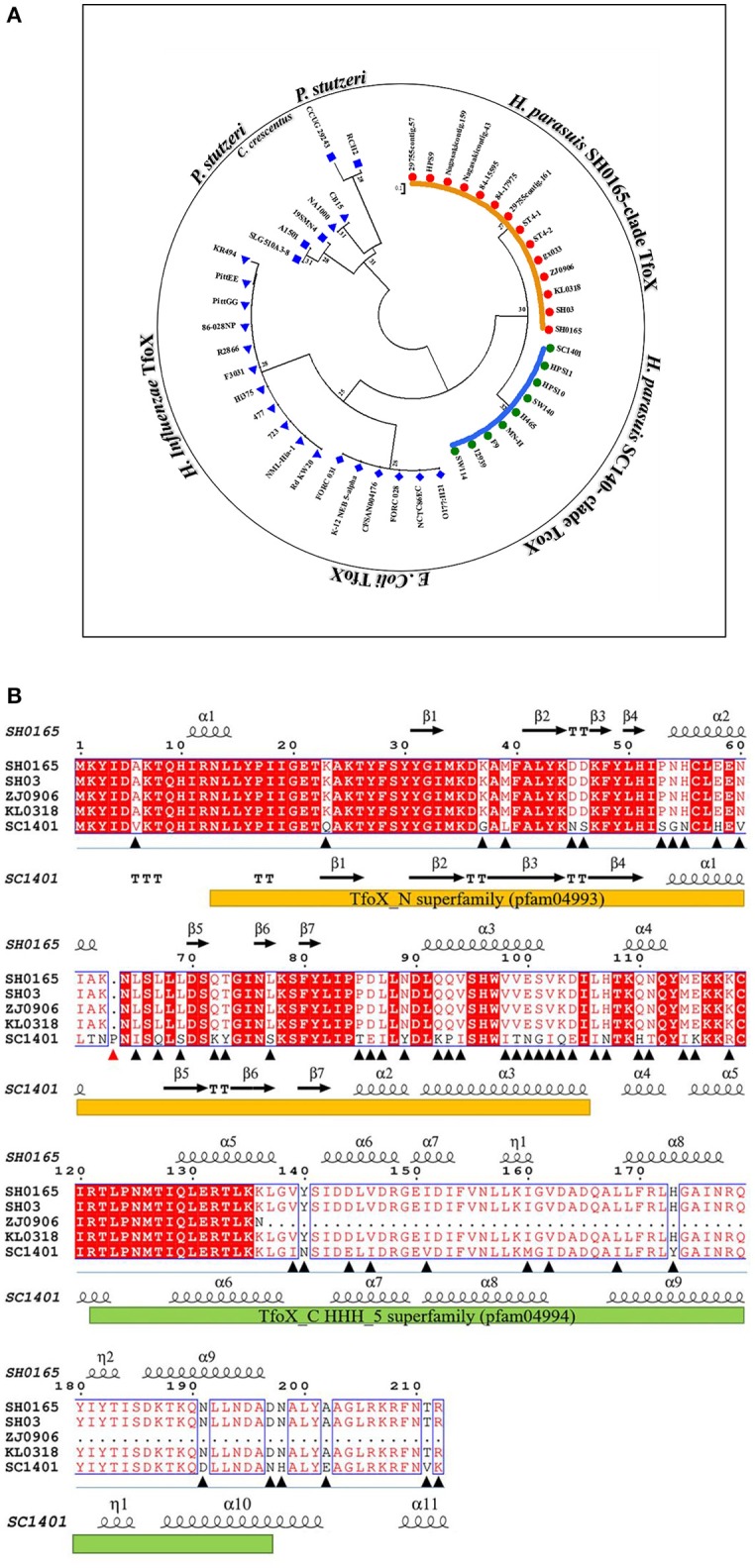
Comparisons of amino acid sequences of TfoX homologs in *H. parasuis*. **(A)** Phylogenetic tree of TfoX in *H. parasuis* (HpTfoX), *E. coli* (EcTfoX), *H. influenzae* (HiTfoX) and in two other outgroups. **(B)** Sequence alignment of TfoX protein in *H. parasuis* SC1401 against four other *H. parasuis* strains using Phyre program version 2.0 and Espript program version 3.0. Orange bar shows N-terminal domain of TfoX; Reseda bar shows C-terminal domain of TfoX; Triangular symbol shows mutation sites of SC1401 TfoX compared to other *H. parasuis* strains (A red one indicates the insertion mutant).

The whole-genomic analysis showed a genome size of 2,277,540 bp of SC1401, with a mean (G+C) content of 40.03%. The total gene number is 2,234, accounting for 87.65% of the whole genome. Comparative genomics analysis (via core-pan gene analysis) demonstrated that SC1401 has 385 strain-specific genes compared to *H. parasuis* SH0165 (CP001321.1), KL0318 (CP009237.1), ZJ0906 (CP005384.1), and SH03 (CP009158.1), which have only 190, 71, 201, and 47 strain-specific genes (dispensable gene), respectively. This may underscore the fact that SC1401 takes up exogenous DNA more readily than the other *H. parasuis* clades (Wang et al., 2014) (SC1401 belong to a different clade compared to the other four strains; Figure 1).

The gene products that are involved in DNA uptake and transformation have been described for *H. influenzae* reference strain Rd KW20 (Maughan and Redfield, 2009b). To identify candidate competence genes in *H. parasuis*, we used BLAST and Rd KW20 sequences to query the genome sequences of *H. parasuis* SC1401 and SH0165, as well as a close-related *A. pleuropneumoniae* strain*-*L20 (which has a low transformation frequency of 1.6^−08^ ± 1.1^−09^) (Bossé et al., 2009) in Genbank (Table 2). A set of CRP-S (non-canonical cAMP-CRP binding sites, see below) regulated competence genes homologs to *H. influenzae* competence genes were identified in *H. parasuis* (MacFadyen, 2000). Some of the homologous sequences may not be involved in DNA uptake, but CRP-S regulated genes, including *radC*, HI0660, HI0365, *ligA*, and HI1631 are considered to be involved in competence (Sinha et al., 2012). Competence genes were divided into three categories according to their functions (Maughan and Redfield, 2009a). Additionally, subcellular locations were predicted with the PSORTb server (http://www.psort.org/) and the functions of gene products were annotated with 6 databases described elsewhere (Kanehisa, 1997; Tatusov et al., 2003; Jungo et al., 2012; Dai et al., 2016), including NR, KEGG, COG, Swiss-Prot, GO and TrEMBL. However, there are no homologs of HI0600 found in *H. parasuis*, and the HI0659 locus found in SC1401 and SH0165 was shown to have a low homology to that of *H. influenzae* Rd. These two genes are annotated as transcriptional regulators, however, their function is still unknown (Sinha et al., 2012).

**Table 2:** CRP-S regulons and competence protein homologs encoded by in *H. parasuis* SC1401 and SH0165 genomes^a^.

**Hin^b^**	**App^c^**	**Hps^c^**					
**Rd KW20**	**L20**	**SC1401**	**SH0165**	**ID to SC1401 (nu)**	**ID to SC1401 (aa)**	**Localization prediction^e^**	**Subject_description**
**REGULATORS**							
*cyaA*(HI0604)	APL_RS05505	A4U84_RS09010	HAPS_RS04830	95	97	Cytoplasmic	Adenylate cyclase, class 1
*icc*(HI0399)	APL_RS10300	A4U84_RS02855	HAPS_RS10275	99	99	Cytoplasmic	Calcineurin-like phosphoesterase family protein
*crp*(HI0957)	APL_RS10410	A4U84_RS03720	HAPS_RS09910	99	99	Cytoplasmic	CRP/FNR family transcriptional regulator, cyclic AMP receptor protein
		A4U84_RS07490	HAPS_RS05700	97	98	Cytoplasmic	
*sxy*/*tfoX*(HI0601)	APL_RS09350	A4U84_RS05295	HAPS_RS07770	74	73	Cytoplasmic	DNA transformation protein and related proteins
**DNA UPTAKE**							
*comA*(HI0439)	APL_RS01005	A4U84_RS02030	HAPS_RS11095	98	99	Cytoplasmic	Competence protein ComA
*comB*(HI0438)	APL_RS01010	A4U84_RS02025	HAPS_RS11100	97	97	Unknown	Hypothetical protein
*comC*(HI0437)	APL_RS01015	A4U84_RS02020	HAPS_RS11105	93	91	Unknown	Chromosome segregation ATPase
*comD*/*pilP*(HI0436)	APL_RS01020	A4U84_RS02015	HAPS_RS11110	95	93	Unknown	Pilus assembly, PilP family protein
*comE*/*pilQ*(HI0435)	APL_RS01025	A4U84_RS02010	HAPS_RS11115	96	98	Outer Membrane	Type IV pilus secretin PilQ family protein
*comF*(HI0434)	APL_RS10620	A4U84_RS05740	HAPS_RS07275	96	98	Cytoplasmic	Phosphoribosyl transferase domain protein
*comE1*/*comEA*(HI1008)	APL_RS07435	A4U84_RS07375	HAPS_RS04120	99	98	Cytoplasmic Membrane	Competence protein ComEA
*pilA*(HI0299)	APL_RS04595	A4U84_RS08530	HAPS_RS09770	98	99	Extracellular/Fimbrial	Prepilin peptidase dependent protein D
*pilB*(HI0298)	APL_RS04590	A4U84_RS08525	HAPS_RS09760	97	99	Cytoplasmic	Tfp pilus assembly pathway, ATPase PilB
*pilC*(HI0297)	APL_RS04585	A4U84_RS08520	HAPS_RS09755	99	100	Cytoplasmic Membrane	Bacterial type II secretion system F domain protein
*pilD*(HI0296)	APL_RS04580	A4U84_RS08515	HAPS_RS09750	99	99	Cytoplasmic Membrane	Tfp pilus assembly pathway, fimbrial leader peptidase PilD
*comN*/*ppdA*(HI0938)	APL_RS10035	A4U84_RS02240	HAPS_RS10900	97	98	Cytoplasmic Membrane	Type II secretory pathway, pseudopilin PulG
*comP*(HI0940)	APL_RS10025	A4U84_RS02230	HAPS_RS10910	98	98	Cytoplasmic Membrane	Function unknown
*comQ*/*ppdC*(HI0941)	APL_RS10020	A4U84_RS02225	HAPS_RS10915	98	97	Cytoplasmic Membrane	Prepilin peptidase dependent protein C
*rec-2*/*comEC*(HI0061)	APL_RS03990	A4U84_RS10535	HAPS_RS02305	99	98	Cytoplasmic Membrane	DNA internalization-related competence protein ComEC/Rec2
HI0365	APL_RS06645	A4U84_RS02730	HAPS_RS10415	99	99	Cytoplasmic	Ribosomal RNA large subunit methyltransferase N
*pilF*(HI0366)	APL_RS06640	A4U84_RS02735	HAPS_RS10405	97	98	OuterMembrane	Fimbrial biogenesis and twitching motility protein PilF-like protein
**TRANSFORMATION**							
*comM*(HI1117)	APL_RS09295	A4U84_RS06900	HAPS_RS08450	99	99	Cytoplasmic	Competence protein ComM
*dprA*(HI0985)	APL_RS09085	A4U84_RS04945	HAPS_RS07640	97	98	Unknown	DNA protecting protein DprA
*radC*(HI0952)	APL_RS10435	A4U84_RS03695	HAPS_RS09940	91	99	Cytoplasmic	DNA repair RadC family protein
*recA*(HI0600)	APL_RS05985	A4U84_RS07560	HAPS_RS05770	94	98	Cytoplasmic	DNA recombination/repair protein RecA
*ssb*(HI0250)	APL_RS04100	A4U84_RS00055	HAPS_RS01845	93	92	Cytoplasmic	Single-strand DNA-binding protein
**UNKNOWN**							
*murE*(HI1133)	APL_RS00065	A4U84_RS01305	HAPS_RS00570	95	97	Cytoplasmic	UDP-N-acetylmuramoylalanyl-D-glutamate−2,6-diaminopimelate ligase
*ligA*(HI1100)	APL_RS06830	A4U84_RS01895	HAPS_RS00055	99	99	Cytoplasmic	NAD-dependent DNA ligase LigA
HI0660	APL_RS07145	×	×			Unknown	Function unknown
HI0659	APL_RS07140	A4U84_RS04905^d^	HAPS_RS01545^d^	55	41	Unknown	HTH-type transcriptional regulator immR
		A4U84_RS04010^d^	HAPS_RS04010^d^	54	41	Unknown	Transcriptional regulator, y4mF family
			HAPS_RS09250^d^	99	100	Unknown	Function unknown
HI1631	×	A4U84_RS08565	HAPS_RS07105	98	98	Cytoplasmic	Restriction endonuclease-like superfamily

a *Hin, H. influenzae; App, A. pleuropneumoniae; Hps, H. parasuis; ID, identity; nu, nucleotide sequence; aa, amino acid sequence; Tfp, transformation pilus*.

b *Gene names with old locus tag*.

c *Homologues with new locus tag*.

d *Homologues in H. parasuis have fairly low identities with that of in H. influenzae, but all annotated to be transcriptional regulators*.

e *prediction run with PSORTb v.3.0. × indicates homologs not found in whole genome*.

Most genes were conserved in both SC1401 and SH0165 with the exception of *tfoX* in view of its vital role in natural competence-regulation. TfoX shares only 73% identity and has unexpectedly evolved into two different clades (SC1401 and SH0165; Figure 1A), indicating a unique heterogeneity in this species in that TfoX is conserved in *E. coli* and in *H. influenzae* (Figure 1A). Intriguingly, blast results using Phyre program version 2.0 and Espript program version 3.0 demonstrated that there are a number of site mutations in SC1401TfoX compared to the four other TfoX (Figure 1B) (Robert and Gouet, 2014; Kelley et al., 2015). A proline residue was found inserted into *H. parasuis* SC1401 TfoX at codon 64th. Moreover, secondary structure comparison showed that SC1401TfoX harbors two more α-helices, indicating that these mutations may affect ultimate functional activity of TfoX protein. We found ZJ0906 possesses four termination codons in ZJ0906 *tfox* gene, Table S3 which results in a truncated TfoX in this strain, indicating that *tfox* gene is not an indispensable gene in bacteria metabolism. In view of the orthology of *tfox* in *H. parasuis*, further studies on whether these molecular diversities lead to the different capacities of natural transformation are needed.

The phenomenon of finding homologs of all known competence genes from *H. influenzae* may be consistent with an ancestral origin of competence in these species (Redfield et al., 2006).

### Competence-related cyclic AMP receptor protein regulons identified in SC1401

**Figure 2:**
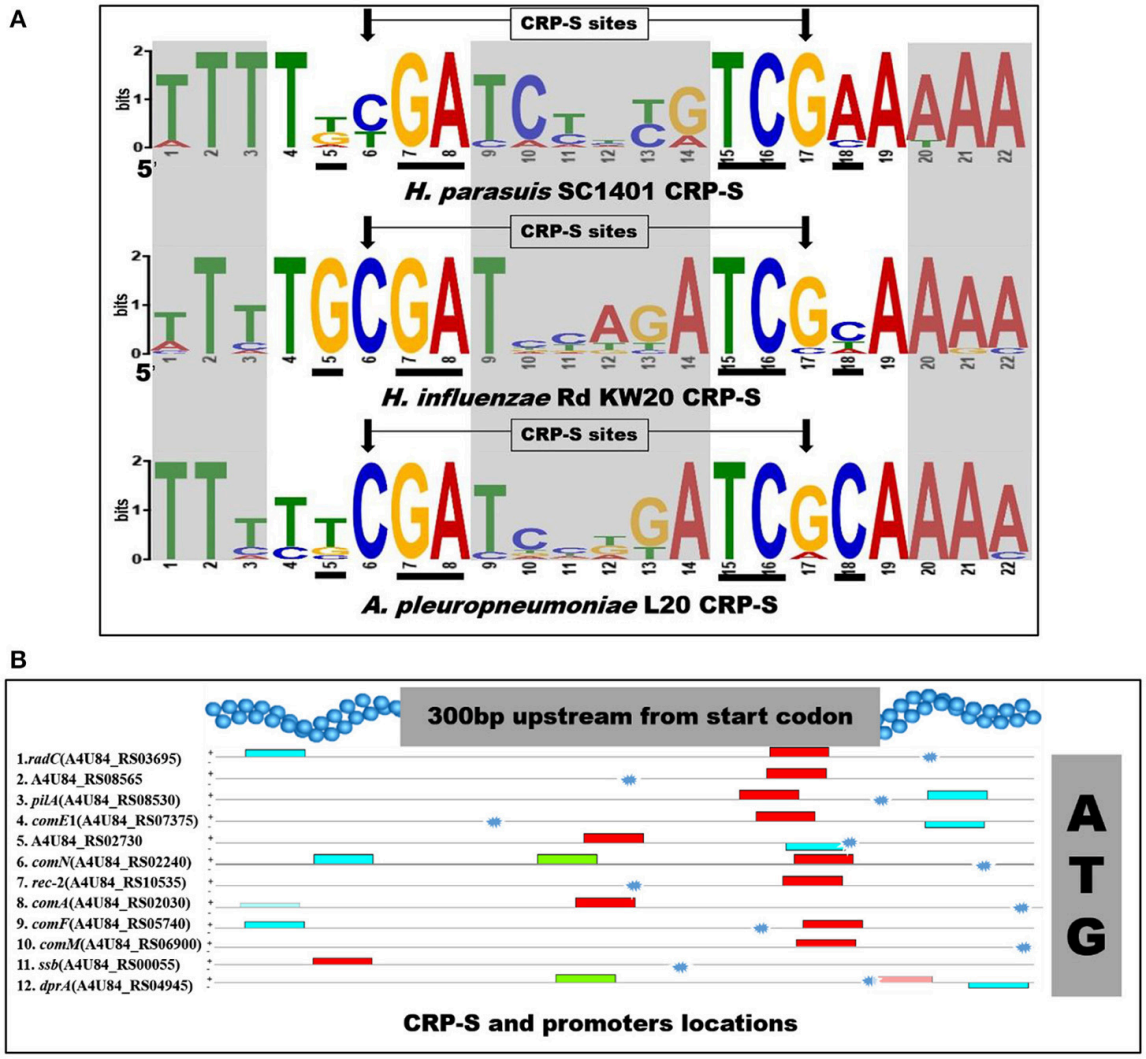
Sequences logos of CRP-S in *H. parasuis* SC1401 and *H. influenzae* Rd KW20 and locations in upstream of 11 competence genes-homologs in SC1401. **(A)** CRP-S logos of 12 *H. parasuis* sites (top, with an *E*-value of 5.3e-035) and 13 *H. influenzae* sites (middle), as well as 15 *A. pleuropneumoniae* sites (bottom). The motifs were found and logos generated with MEME (http://meme-suite.org/tools/meme) (Bailey and Elkan, 1994). Bases important for cAMP-CRP-DNA binding reported by Prof. Redfield are shown in black underlines, and the characteristic positions of CRP-S sites (C_6_/C_17_) are shown in arrows. Noncore sites are shaded. Logo of CRP-S of *H. influenzae* was reproduced with Prof. Redfield's permission. **(B)** CRP-S locations in 12 *H. parasuis* competence genes. Red boxes indicate 22 bp consensus sequences with high similarity with CRP-S shown in Table 3 and Figure 2 (top); the green and blue ones indicate similar motifs. Note, the CRP-S motif predicted in promoter region of *dprA* was partly homologous, thus indicated by a semitransparent red box. Promoter of each gene is shown with explosion icon, which was predicted with BPROM (http://linux1.softberry.com/berry.phtml).

In *H. influenzae*, multiple Sxy-dependent *c*yclic AMP *r*eceptor *p*rotein (CRP, or catabolite activator protein) regulons are required for transformation (Cameron and Redfield, 2008; Sinha et al., 2012). The CRP-S (cAMP-CRP complex binding sites; competence regulons; previously named CRE) is a promoter associated 22-bp competence regulatory element (CRE). This competence-related sequence/site was determined to be a new CRP binding site under Sxy-regulation (the ortholog of *tfox* in *H. parasuis*) (Redfield et al., 2005). Thus, CRP-S regulon was regulated by CRP-cAMP complex and functions in natural competence/transformation (Cameron and Redfield, 2008). Notably, the CRP-S differs from canonical (CRP-N) sites in that the former one requires both the CRP and Sxy/TfoX proteins for transcription activation and the 6th base is cytosine, instead of thymine (Cameron and Redfield, 2008). In our study, we found a highly similar consensus sequence located in the promoter regions of 12 competence homologs in *H. parasuis* SC1401 (Figure 2 and Table 3). These “un-strict” reverse complement consensus sequence shows high identity to the *H. influenzae* and *A. pleuropneumoniae* CRP-S consensus sequences and retains almost all of the bases from the CRP-DNA binding sites/residues (Redfield et al., 2005; Bossé et al., 2009) (Figure 2A). Noncore sites display a high degree of sequence diversity (heterogeneity), indicating interspecific differences among these species. Of note, further inspection of the promoter region of *dprA* found a suboptimal (*p*-value of 4.46e-8) CRP-S motif for it is more variable in certain sites (Figure 2B). No direct evidence has been found that CRP and TfoX/Sxy could bind to CRP-S in *H. parasuis*. However, it has been reported that CRP binds to cAMP and form CRP-cAMP complex (Cameron and Redfield, 2008). Further studies to clarify the function and/or the interaction of CRP-S and CRP/TfoX/Sxy in *H. parasuis* are needed.

**Table 3:** CRP-S (CRE) promoter sites identified in 11 competence genes in *H. parasuis* SC1401.

	**Name**	***P*-value**		**CRP-S Sites**	
**1**.	***radC*** **(A4U84_RS03695)**	**3.04e-12**	**AACGCCCTAT**	**TTTTTGCGATCTTTGTCGAAAAA**	**AGAGAAAAAG**
**2**.	**A4U84_RS08565**	**1.35e-12**	**AAAAAATAAA**	**TTTTTCGATCTCTGTCGAAAAA**	**TTAAAAGAAT**
**3**.	***pilA*** **(A4U84_RS08530)**	**5.39e-12**	**AAAAAACGAT**	**TTTTTCGATCTACGTCGAAAAA**	**ATAAATGTTG**
**4**.	***comE*****1 (A4U84_RS07375)**	**1.85e-12**	**ATTTTTCAAT**	**TTTTTCGATCTGCGTCGAAAAA**	**CACATTTTTA**
**5**.	**A4U84_RS02730**	**5.39e-12**	**GCCGAACTAT**	**TTTTGTGATCCCCGTCGAAAAA**	**TTCATTATTT**
**6**.	***comN*** **(A4U84_RS02240)**	**1.08e-11**	**ATCTTTCTAA**	**TTTTTCGATCTATGTCGAAAAA**	**ATAAAAATTG**
**7**.	***rec*****-2 (A4U84_RS10535)**	**1.08e-11**	**CAATATTTAT**	**TTTTGTGATCCCTGTCGAAAAA**	**CATTATTTTT**
**8**.	***comA*** **(A4U84_RS02030)**	**1.77e-10**	**TAAATATGAT**	**TTTTACGATCTTCATCGAAAAA**	**TTCTCTTTTG**
**9**.	***comF*** **(A4U84_RS05740)**	**1.62e-10**	**ATTTTACTCT**	**TTTTTCGATCCGTGTCACAAAA**	**ATTCCCCAAA**
**10**.	***comM*** **(A4U84_RS06900)**	**7.69e-10**	**CTTTAACTAT**	**TTTTGTGACACTCGTCGAAAAA**	**CCAACATTTT**
**11**.	***ssb*** **(A4U84_RS00055)**	**7.55e-9**	**AAATTTTCAA**	**ATTTTCGATCATTATCGCATAA**	**ATTGATTAAC**
**12**.	***dprA*** **(A4U84_RS04945)**	**4.46e-8**	**AACGATGAAA**	**ATTTTCTACCTCCAACAAAGAA**	**AAAGTTAAAG**

*CRP-S motif was found in 300 bp upstream of all 12 genes in H. parasuis SC1401. P-value represents the probability that an equal or better site could be found in a random sequence of the same length conforming to the background letter frequencies. Bold and color values indicate flanking regions and their corresponding candidate CRP-S site in the promoter region of different competence gene. Note, the CRP-S motif of dprA is more variable (p-value of 4.46e-8), thus was identified manually*.

### Natural transformation is stable in *H. parasuis* SC1401

**Figure 3:**
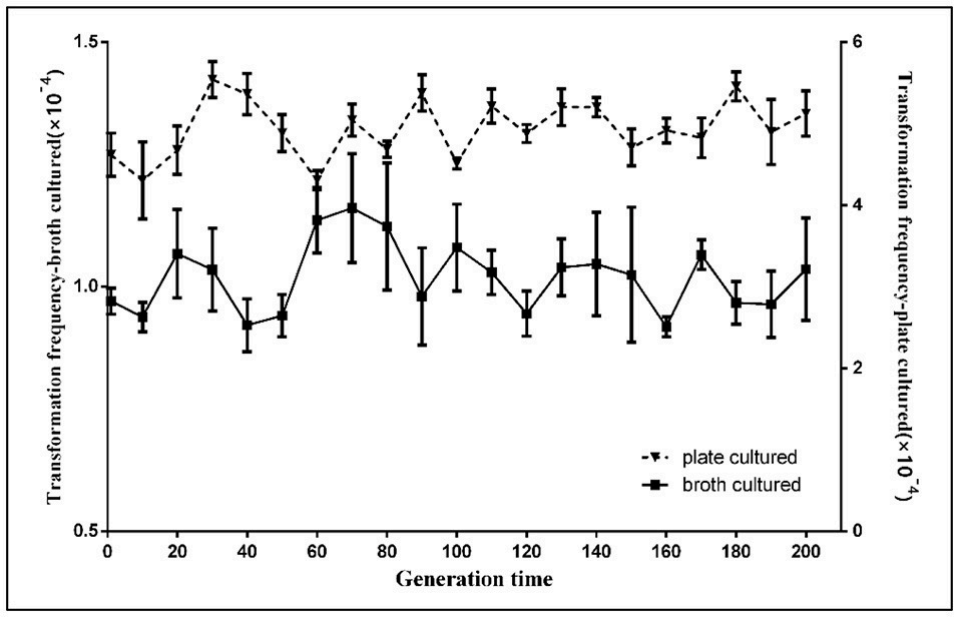
Natural transformation stability analysis of *H. parasuis* SC1401 after continuously passaging. The natural frequencies of every 10 generations of *H. parasuis* SC1401 were determined through TSB++ transformation methodology (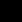) and TSA++ transformation methodology (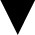). The natural transformation frequency was determined from the number of antibiotic-resistant cfu mL^−1^ divided by the total cfu mL^−1^ scored on non-selective agar. Data points represent the mean values of three replicates, and error bars indicate standard deviations.

A previous report found that *Acinetobacter baylyi* ADP1 transformability was reduced after 1,000 generations for the new InDels of IS*1236* transposable elements and other large-fragment deletions on the chromosome (Renda et al., 2015). We studied the stability of natural competence in *H. parasuis* SC1401. After the parent bacteria SC1401 were serially passaged (from F1-F200), the natural transformation frequencies of different generations of SC1401 were analyzed. In each generation, both broth-cultured (in TSB++) and plate-cultured (13 h on TSA++) bacteria were transformed with genomic DNA of SC1401Δ*htrA*::kan. Results demonstrated negligible changes in transformation frequencies with both methods (Figure 3), which indicates that natural transformation is stable within a short-term passage in this species. This also suggests that natural transformability varies greatly in different species due to different competence regulatory elements/circuits and/or environmental cues of triggering competence (Krüger and Stingl, 2011; Seitz and Blokesch, 2013; Hülter et al., 2017).

### Natural transformation is induced at the onset of early log-phase and reaches peak level in stationary phase in *H. parasuis*

**Figure 4:**
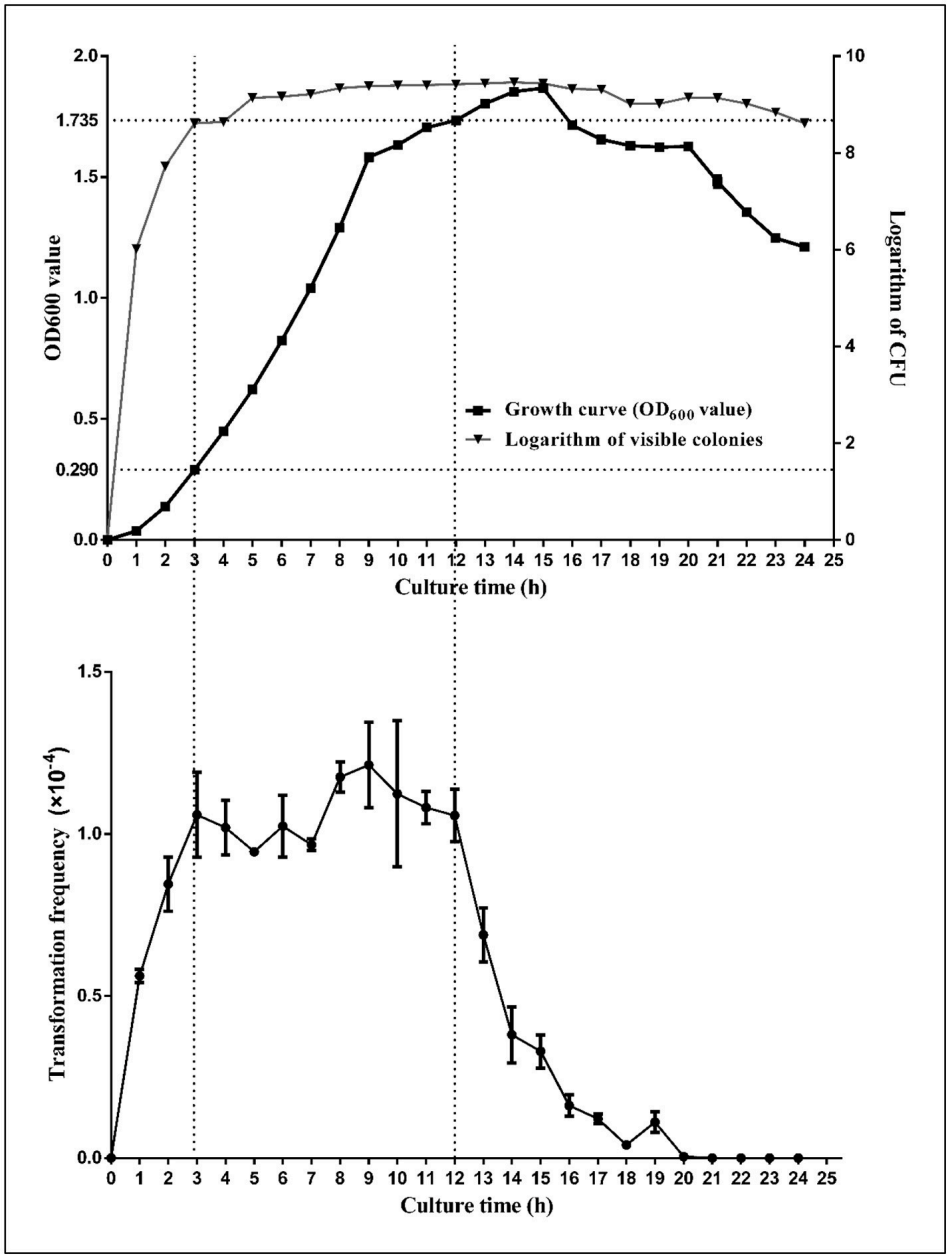
Growth Curve of SC1401 and transformation frequencies analysis of bacteria cultured in TSB++. The growth curves of the *H. parasuis* SC1401 (top; “
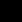
” indicate growth curve; “
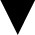
” indicate cfu by plate counting method) and transformation frequencies of bacteria in different growth phase (bottom). Bacterial growth was monitored by measurement of optical density at 600 nm and at each time point (from early logarithmic phase to decline phase along the whole growth curve), the natural transformation frequency was determined from the number of antibiotic-resistant cfu mL^−1^ divided by the total cfu mL^−1^ scored on non-selective agar. Data points represent the mean values of three replicates, and error bars indicate standard deviations.

In our study, *H. parasuis* cultures demonstrated an increasing transformability with the growth of bacteria during the log-phase, reaching peak competence level at the onset of stationary-phase; the bacteria maintained high competency levels from early- to mid-stationary phase (OD_600_ from 0.290 to 1.735) (Figure 4). However, when cultures reach the decline phase (OD_600_ from 1.735 to 1.625), competence levels decreased significantly (nearly one-quarter of peak level, *P* < 0.01). Our findings are, to some extent, similar to those from early reports on the time of peak transformability in *H. influenzae*, but demonstrate some differences between those of *A. baylyi BD413, S. pneumoniae* and *B. subtilis*, whose competence are at a short time or develop at the onset of stationary phase (Juni and Janik, 1969; Juni, 1978; Johnsborg et al., 2007).

The transformability of *H. influenzae* has been studied for decades, and the natural transformation mechanism of this species has been well-characterized, despite some gaps in knowledge. It has been confirmed that *H. influenzae* become moderately competent as growth slows during late-log phase in rich medium, however, when log-phase culture is transferred to defined competence medium (M-IV), the cells become maximally competent (Herriott et al., 1970), which is a somewhat different pattern than that of *H. parasuis*, which is more easily induced into a naturally competent state even in the well-nutritive TSA++/TSB++(Zhang et al., 2015). Both species belong to the family of *Pasteurellaceae* in γ-proteobacteria (Xu et al., 2011), which may share some common regulators/signaling circuits as described above and yet still have interspecific differences regarding DNA uptake/incorporation systems or cues of triggering natural transformation. In the light of these findings, we conclude that the *H. parasuis* natural transformation circuit is linked to growth status and may already be enabled in the early log-phase period, but shows peak level when bacteria enter stationary phase.

### Natural transformation in *H. parasuis* shows a phenomenon of concentration-dependency

**Figure 5:**
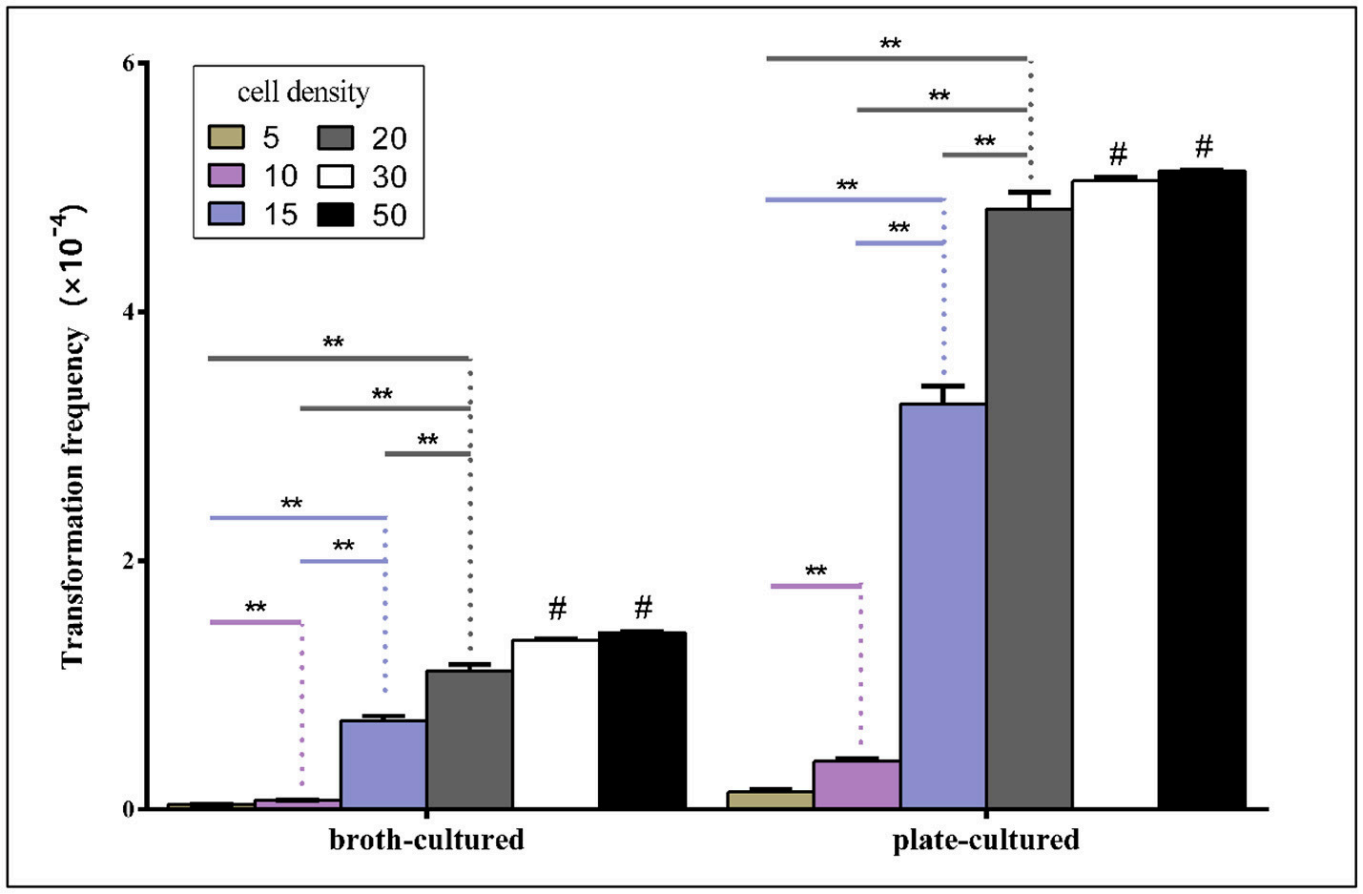
Transformation frequencies analysis by two methods using different cell density. Competency rates vs. cell suspension density (measured by OD_600_-value) were tested using six different cell densities. Suspensions were made of bacteria grown on TSB++/TSA++. Transformation frequencies were calculated as described above. Histograms represent the mean values of three replicates, and error bars indicate standard deviations. “^**^” means significant difference between groups at 0.01 level. “^#^” means the difference of transformation frequency is not significant in groups of cell concentrations of 20 and 30/50, respectively by LSD method.

To evaluate whether the density of bacteria spotted on TSA++ prior to adding donor DNA could influence natural transformation, suspensions of varying densities were made of bacteria grown on both TSB++ and TSA++− measured by OD_600_ with a 10-fold or a 100-fold dilution and routine transformation experiments were performed. As illustrated in Figure 5, the natural transformation frequencies were positively correlated with bacterial concentrations in both methods when OD_600_-value is within 20. However, the transformation frequency demonstrated a significant jump when bacterial concentrations were raised from OD_600_ = 10 to 15 in both methods, suggesting an unknown concentration-related molecular mechanism may play an important role in regulating natural transformation in this species. Transformation frequencies of high density bacterial suspension (calculated by OD_600_) higher than 20 are not significant (*P* < 0.01 level) compared to the highest transformation frequencies. Optimum competency rates were obtained when recipient bacteria were at an OD_600_ of ~20 (Figure 5).

### *H. parasuis* preferentially takes up homologous DNA compared with DNA from heterologous species

It has been well established that *H. influenzae, N. meningitides*, and *N. gonorrhoeae* preferentially take up DNA from closely related species (Johnsborg et al., 2007). *Campylobacter jejuni* discriminates between unmethylated/methylated DNA (Beauchamp et al., 2017). To determine the preference of DNA by *H. parasuis* cells, we examined whether *H. parasuis* could take up heterologous DNA and further performed competition uptake experiments.

**Figure 6:**
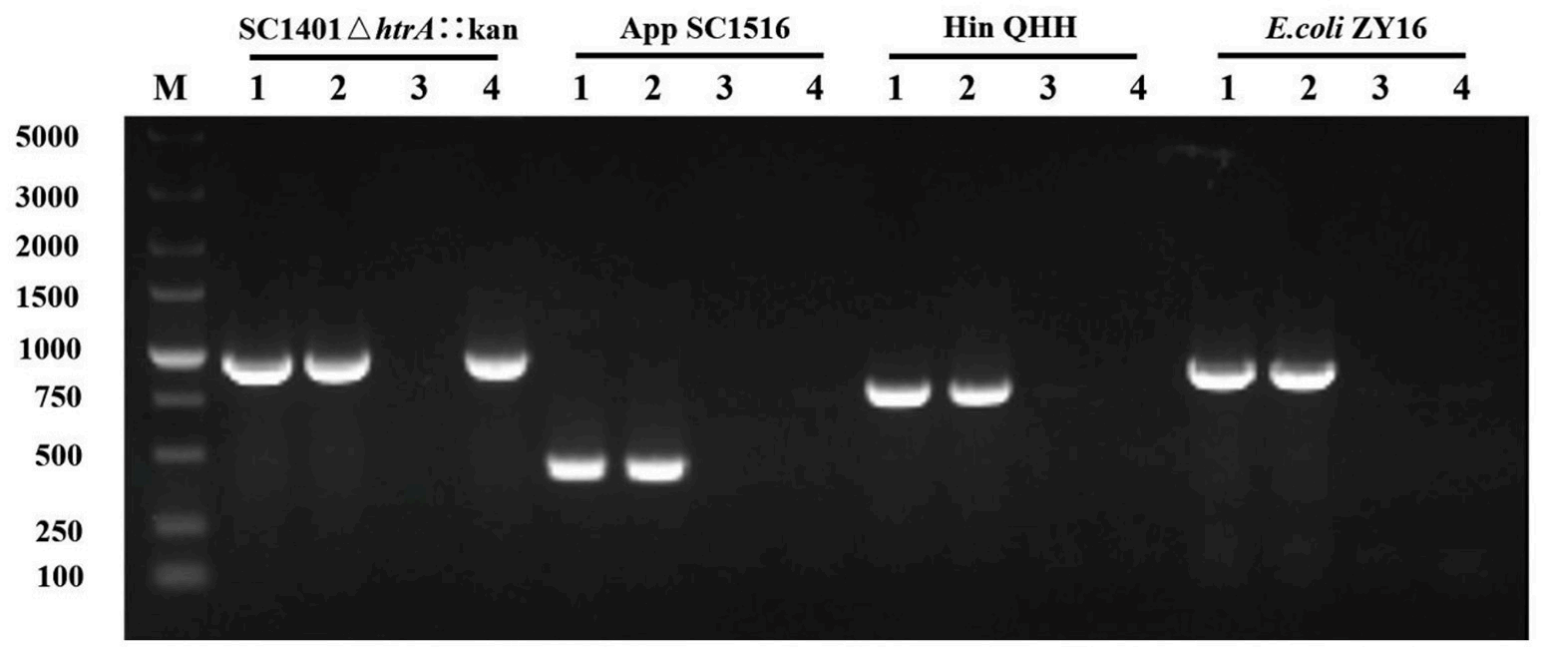
*H. parasuis* can take up heterologous DNA but degrade it after the bacteria was passaged for one generation. The 16S rDNA of different donor genomic DNA to transform wild type *H. parasuis* SC1401 was examined by PCR. Kan-cassette of homologous derivative SC1401Δ*htrA*::kan was examined as a control. Lane 1: Respective genomic DNA (positive control). Lane 2: Pellet. Lane 3: Dnase-treated supernatant. Lane 4: Passaged bacteria. M: DNA ladder (DL5000, TaKaRa, Japan).

To study if DNA from other species could be taken up by *H. parasuis* SC1401, routine natural transformation on TSA++ of *H. parasuis* SC1401 with different DNA (1 μg) was performed. After the mixture was incubated on TSA++ at 37°C for 5 h, the extracellular DNA was degraded by DnaseI. PCR runs to amplify 16S rDNA were used to determine the presence or absence of the donor DNAs in the supernatant and in the recipient bacteria (pellet), as well as in the passaged bacteria. Here we show the DNA uptake results of purified genomic DNA from *A. pleuropneumoniae, H. influenza*, and *E. coli* in transforming *H. parasuis* SC1401. As shown in Figure 6, all 16S rDNAs could be obtained from a Dnase-treated *H. parasuis* SC1401 pellet after the mixture were incubated for 5 h on TSA++, but not from supernatant, which indicated that these DNAs could be uptaken by *H. parasuis* SC1401. However, 16S rDNAs of heterologous species could not be detected after recipient bacteria were passaged for one generation in TSB++. In contrast, when we used *H. parasuis* SC1401Δ*htrA*::kan as a control, a Kan-cassette was still present after the recipient bacteria were passaged. As a control, corresponding 16S rDNA of each species without Dnase treatment could still be amplified from their supernatant (data not shown). These results demonstrate that *H. parasuis* SC1401 could take up heterologous DNA, which is independent of methylation of these DNA, but the DNA was degraded after several passages.

**Figure 7:**
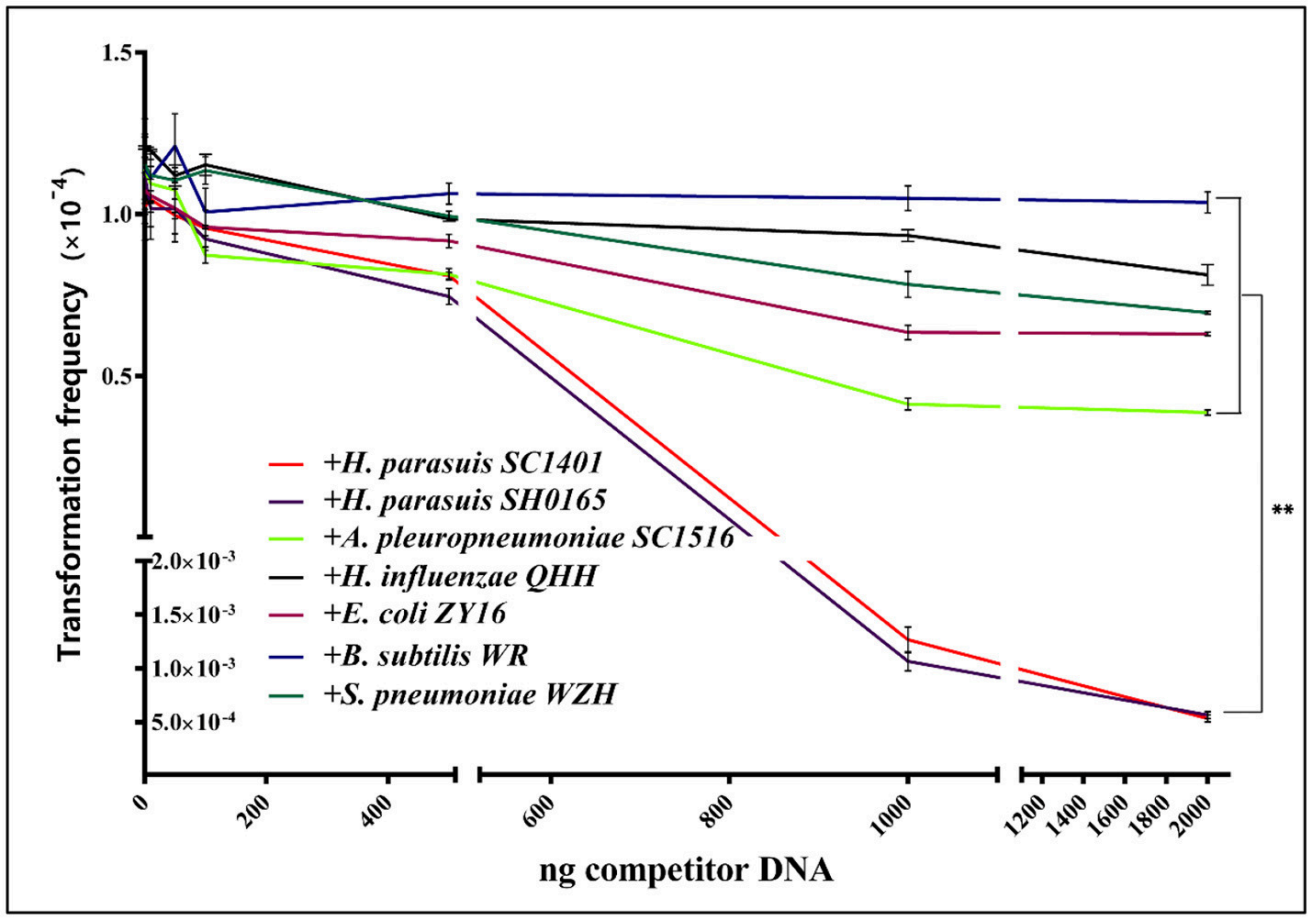
*H. parasuis* transformation could be inhibited with unmarked homologous DNA. *H. parasuis* transformation could be inhibited by exogenous DNA and moderately inhibited by DNA from closely-related *A. pleuropneumoniae*. Increasing amounts (0, 10, 50, 100, 500, 1,000, and 2,000 ng) of competitive DNA from either homologous and heterologous strains were added to the mixture with constant amount (1 μg) of SC1401Δ*htrA*::kan chromosomal DNA. Transformation frequency shows the number of Kan^R^ transformants per cfu of recipient cells. Data are representative of three independent experiments. Solid lines with different colors indicate source of competitor DNA.

To further study if *H. parasuis* preferentially take up homologous DNA, wild-type *H. parasuis* SC1401 was transformed with constant amount (1 μg that is efficiently transformed) of DNA from SC1401Δ*htrA*::kan and increasing amounts of purified DNA from homologous wild-type *H. parasuis* strains SC1401 and SH0165, *H. influenzae, A. pleuropneumoniae* strain SC1516 (both belong to *Pasteurellaceae*), *E*. *coli* strain ZY16, as well as two far-related Gram^+^ bacteria: *B. subtilis* strain WR, and *S. pneumoniae* strain WZH, respectively. The transformation frequencies were demonstrated in Figure 7. The competitive assay results show that the addition of 1 μg or more of unmarked homologous DNA from *H. parasuis* SC1401 and SH0165 significantly suppresses the transformation frequencies of SC1401Δ*htrA*::kan DNA. Additional amounts of heterogeneous DNA were not able to effectively compete SC1401Δ*htrA*::kan DNA as expected. However, we observed that DNA of *A. pleuropneumoniae* (a closely related species to *H. parasuis*) had a moderately competitive effect relative to DNA derived from other unrelated organisms (Xu et al., 2011). The results indicate that *H. parasuis* has a specific mechanism for differentiating homologous DNA of closely related species from DNAs of other organisms, but preferentially takes up homologous DNA. We postulated that a biased species-specific DNA uptake signal sequence (USS or DUS), which has been reported in *H influenzae* and in *Neisseria*, may play an important role in HGT. Although *A. pleuropneumoniae* (App) has the same 9-mer USS: 5′-ACCGCTTGT that promotes natural transformation in these species (Redfield et al., 2006; Zhang et al., 2012), and these USSs were abundant in the genome of App (more than 500 copies), its genomic DNA demonstrated only moderately competitive effects on genomic DNA of *H. parasuis*. Other studies showed conflicting results concerning the effect of USS (*Apl* subclade 5′-ACCGCTTGT-3′) in this species (Bigas et al., 2005; Zhang et al., 2014; Li J. et al., 2016). The exact molecular markers or restriction modification system (R-M) governing this process remain to be identified.

### TSA+ + −cultured *H. parasuis* show significant higher natural transformation frequency than the TSB+ + −cultured

**Figure 8:**
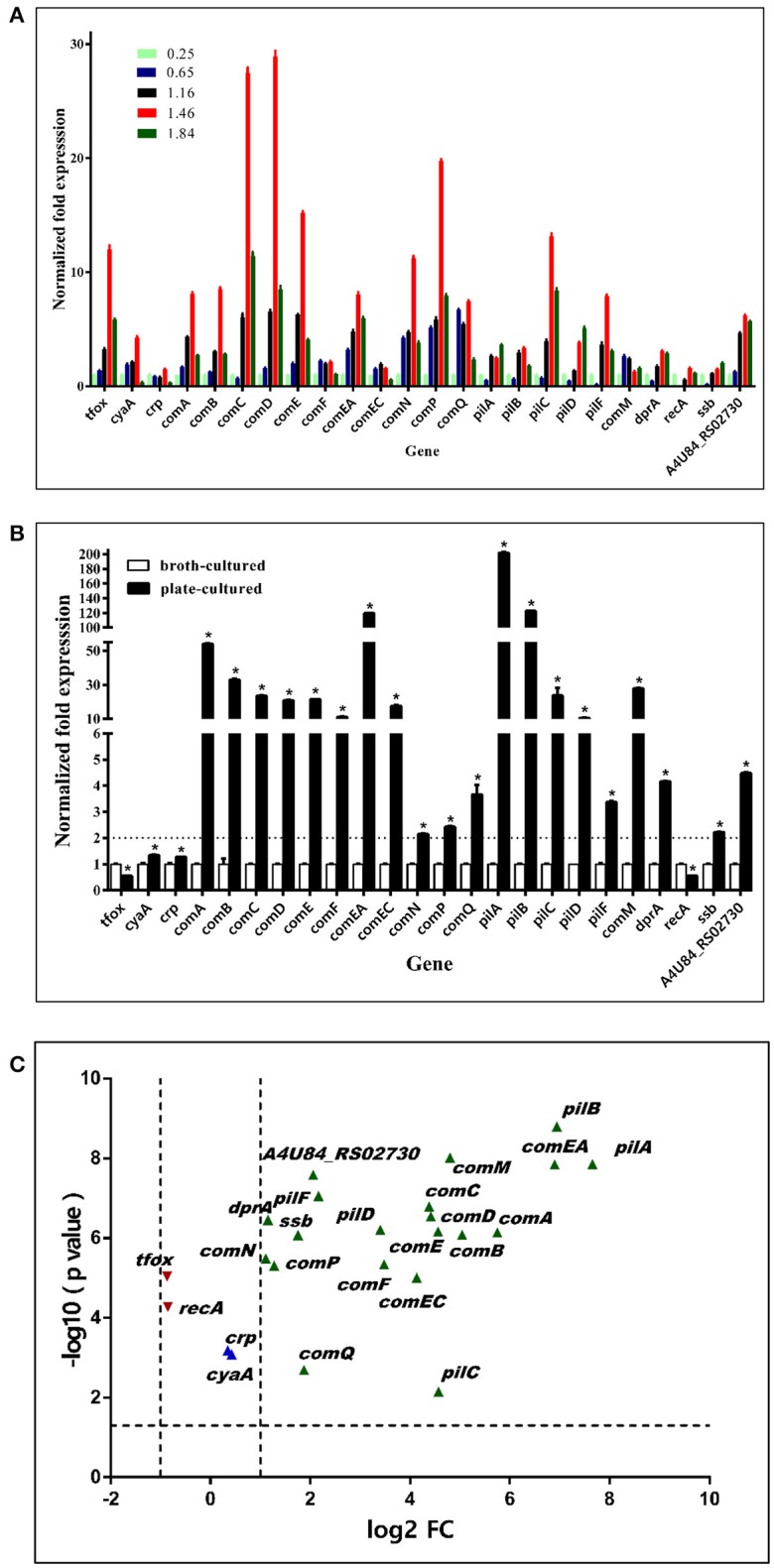
Expression levels of competence genes of bacteria cultured in TSB++ and on TSA++. **(A)** qRT-PCR analysis of mRNA levels of candidate competence genes in *H. parasuis* SC1401in different growth phase measured by optical density at 600 nm. **(B)** qRT-PCR analysis of mRNA levels of *H. parasuis* SC1401 cultured in TSB++ and on TSA++. **(C)** Volcano plot of gene express levels of bacteria cultured on TSA++ compared to that in TSB++. Horizontal dotted line indicates significance test result and vertical dotted lines indicate fold changes. The data represent means standard errors (*n* = 3), and error bars indicate standard deviations. Asterisks indicate statistical significance using one-way ANOVA (*P < 0.05).

In an independent experiment, we found that bacteria grown on TSA++ for 13 h prior to transformation experiments could reach the highest natural transformation level (data not shown). Time-dependent gene expression profiles of multiple candidate transformation regulons and uptake loci were further analyzed. As illustrated in Figure 8A, the vast majority of genes had the highest expression levels when bacteria were harvested at an OD_600_ = 1.46 (about 2.4 × 10^9^ cfu/mL), a stationary phase measured by visible colonies calculation (Figure 4). Therefore, we compared the expression levels of these candidate natural competence-related genes in bacteria cultured in TSB++ (OD_600_ = 1.46) and on TSA++ (13 h). Among the 24 genes, 20 genes were significantly up-regulated more than two times when bacteria were cultured on TSA++ (*P* < 0.05) (Figures 8B,C). *comA, comEA, pilA*, and *pilB* were the most highly up-regulated genes on TSA++ (more than 50-fold, Figures 8B,C). Taken together, bacteria cultured on TSA++ reach a significantly higher natural transformation level than when cultured in TSB++, despite observing the highest expression levels of these genes in the later method. This phenotype is, largely, compatible with gene expression level in this species, but the cues responsible for up-regulation of these genes still need to be identified.

### *H. parasuis* clinical isolate SC1401 shows significantly higher gene expression levels of candidate competence regulons than non-transformable strain SH0165

**Figure 9:**
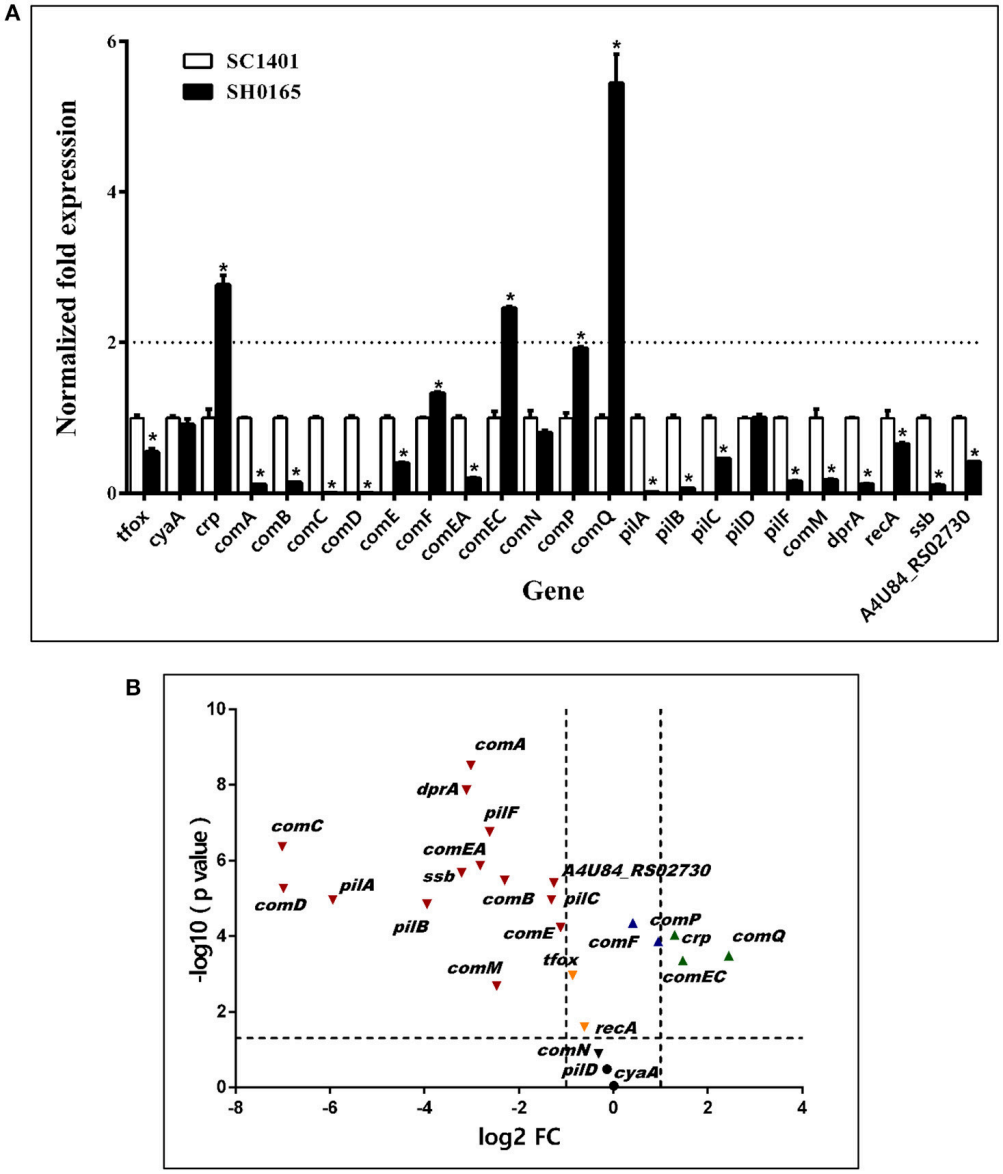
Expression levels of competence genes of *H. parasuis* SC1401 and SH0165 **(A)** qRT-PCR analysis of mRNA levels of *H. parasuis* SH0165 and SC1401. **(B)** Volcano plot of gene express levels of *H. parasuis* SH0165 compared to those of SC1401. Horizontal dotted line indicates significance test results and vertical dotted line indicates fold changes. The data represent means standard errors (*n* = 3), and error bars indicate standard deviations. Asterisks indicate statistical significance using one-way ANOVA (*P < 0.05).

*H. parasuis* reference strain SH0165 (Genbank ID: NC_011852.1) was found to be a non-naturally competent strain *in vitro*. Thus, a qRT-PCR analysis was performed to compare the transcription levels of multiple candidate competence genes in SH0165 and SC1401. As illustrated in Figure 9, there were only three genes (*crp, comEC, comQ*) in SH0165 that showed much higher expression levels than in SC1401 (*P* < 0.05), however, there were 14 genes with significantly lower expression levels than in SC1401 (Figure 9B). The expression levels of candidate uptake components *comD, comC*, and *pilA* were nearly 130-, 123-, and 40-fold lower than in SC1401, respectively (Figure 9A). The differences in *cyaA, comN*, and *pilD* were not significant (*P* > 0.05). These results revealed that the highly naturally competent strain SC1401 up-regulated most of the candidate natural competence-related regulons/components.

## Discussion

The function of natural competence of bacteria is still not completely understood. The predominant view posits that competence promotes bacterial adaptability to environment, serving to accelerate selection rates for bacteria growing in a specific niche (Dorer et al., 2011). A typical example is that *Helicobacter pylori* responds to DNA damage with an increase in competence levels. The resulting increase in exogenous DNA uptake presumably helps the bacteria to acquire traits selected for survival in a harsh gastric environment (Dorer et al., 2010, 2011; Wroblewski et al., 2010). Other viewpoints about functions of competence include uptake of DNA as a nutrient source in otherwise nutrient-poor conditions. The consequence of defined starvation M-IV medium inducing high transformability in *H. influenzae* is consistent with the natural habitat in upper respiratory tract with limited nutrients in this species (Herriott et al., 1970; Seitz and Blokesch, 2013). Likewise, it has been reported that starvation medium M-IV can also promote natural transformation in *H. parasuis* (Li J. et al., 2016), but the details of the mechanism are not clear as those in *H. influenzae*. Lorenz and Wackernagel (1991) demonstrated that limiting carbon, nitrogen, and phosphate resources stimulated the transformation in *P. stutzeri* (Lorenz and Wackernagel, 1991). Palmen et al. (1994) concluded that “the biological function of natural transformation in *A. calcoaceticus* is not to provide the cell with nutrients” and that ‘the regulation of competence development as a function of the stringency of carbon, nitrogen, and phosphate limitation’ was just opposite to what one would expect if the “nutrient supply hypothesis” would apply. However, in *H. parasuis*, the molecular mechanism and cues inducing a high transformability have not all been elucidated. We find only 11% (5/45) of *H. parasuis* strains are naturally competent; this may be due to the use of our screening methods in transformation assay that are not sensitive enough or possibly because some bacteria only become competent *in vivo*. Whole genome sequencing (WGS) of the highly competent SC1401 strain may, to some extent, help to fill in the numerous gaps in our knowledge of *H. parasuis* competence factors (Dai et al., 2016).

Based on above findings, we further investigated the stability and induction phase of natural competence in SC1401. The results showed that competence in this organism was stable upon a 200 generation passage; natural transformation can be triggered in the early-log phase of growth and reaches a peak level during late-log phase growth stage until the mid-stationary phase, where competence begins to diminish significantly. This is a very different pattern from that seen in some other bacterial species. In *H. pylori*, for instance, the most efficient transformation of this species occurs when DNA is introduced prior to exponential phase (Israel et al., 2000), indicating that natural transformation regulatory pathways or environmental cues triggering competence may be different in different bacterial species/genera.

High cell concentrations were found to promote higher transformation rates. We assume that this phenomenon may be due to high cell density inducing quorum sensing (QS). QS has been well studied in *Vibrio cholerae*, where a HapR-dependent high transformation rate is mediated by a high recipient bacterial density and upregulation of major competence proteins *comEA* and *comEC* (Jobling and Holmes, 1997; Whatmore et al., 1999; Wang et al., 2011). A homolog of HapR has not yet been identified in *H. parasuis*. However, we found another two candidate quorum signaling and sensing genes in SC1401: *luxS*, an enzyme to generate DPD (4,5-dihydroxy-2,3-pentonedione); *rbsB*, a periplasmic binding protein which functions as part of an ABC transporter for ribose sugars that has been reported to be an AI-2 (autoinducer-2) uptake regulator (Waters and Bassler, 2005). Neither these nor their analogs have been investigated in *H. parasuis* and researchers have yet to determine if these specific loci exert an influence on competence rates.

DNA uptake signal sequences (USS) were reported to be located on transforming DNA fragments, which allow such fragments to be readily taken up by hosts. As USS or DUS is required in genetic transformation in *H. influenzae* or *Neisseria* spp. in which these motifs increase genetic transformation frequencies by 100- to 10,000-fold, and a mismatch of conserved 3 to 4 bp in USS or DUS of distinct exogenous sequences with multiple “dialects” significantly reduce DNA uptake. These two species are apparently not transformable with DNA from other unrelated bacterial clades. We speculate that these two species benefit by evolving mechanisms for stringent limitations on DNA uptake. Competitive transformation was still found in *P. stutzeri*, but no similar USS or DUS has been reported (Carlson et al., 1983). Homology-facilitated illegitimate recombination was much less efficient in these species (Meier and Wackernagel, 2003). Whereas in *S. pneumoniae* and *Acinetobacter*, no species-specificity has been observed with respect to DNA uptake. In our study, we found *H. parasuis* still has the ability to discriminate homologous DNA from heterogeneous DNA. The marked DNA could be competitively inhibited with regard to uptake, which may be followed by homologous recombination. Unlike in *H. influenzae*, the recognition of incoming DNA in *H. parasuis* is not that tightly regulated as indicated by the moderate inhibition effect from *A. pleuropneumoniae* DNA, and DNA of *E. coli* slightly inhibited DNA transformation in *H. parasuis*. The precise mechanism of exogenous DNA discrimination in *H. parasuis* has yet to be elucidated, but it appears that this species has a less stringent competence control mechanism than the ones found in *H. influenzae* and *Neisseria* spp. Specially, DNA from wild-type *A. pleuropneumoniae* moderately inhibited the host DNA of *H. parasuis*. The *A. pleuropneumoniae* genome bears more than 500 copies of USS as in *H. parasuis*, but couldn't effectively outcompete *H. parasuis* genomic DNA in uptake. These discrepancies suggest that the USSs that play an important role in DNA uptake in this species or other restriction barriers tightly regulating natural transformation are still to be identified (Li J. et al., 2016).

It has been well established that an array of competence genes has been involved in natural transformation regulation. The standard *H. influenzae* strain Rd KW20 and a closed-related species *A. pleuropneumonia* provides a reference point for identifying candidate regulons in *H. parasuis*. We found intact homologs of competence genes in this species and several competence-related cyclic AMP receptor protein regulons that located in the promoter regions of almost 12-competence homologs (when the suboptimal CRP-S of *dprA* was also taken into consideration; Figure 2B). We compared their expression profiles between TSB++/TSA++ cultured bacteria and between the highly transformable strain SC1401 and a non-transformable strain SH0165, the results demonstrated that most of these candidate competence genes are significantly up-regulated on bacteria pre-cultured on TSA++ and in SC1401. This is largely compatible with natural competence phenotype. However, it is notable that a core competence regulator Tfox, an ortholog of Sxy in *H. influenza* and Tfox^*VC*^ in *Vibrios*, shares only 73% identity between SC1401 and the other clade in *H. parasuis*. As this is a vital transcription activator of competence development in many bacteria, *tfoX* is highly conserved in these species. However, Tfox can be divided into two clades in *H. parasuis*. Further analysis of the RNA secondary structures shows that these two clades of *tfox* have different topological structures but an identical Shine-Dalgarno sequence (SD sequence) AGGU could be found in both promoter regions of *H. influenzae sxy* and in the same sites of *H. parasuis* SC1401. The promoter region of SH0165*tfox* RNA lacked this particular SD sequence. Tfox proved to be an indispensable factor in regulating natural transformation in *H. parasuis* SC1401 in an independent experiment. We found SC1401Δ*tfox* completely lost its natural transformability (data not shown). Whether the molecular diversity of TfoX, as stated above, is one of the factors that result in different transformabilities in this species is unknown. Further study to address this important issue is needed. In addition, *pilA, pilB* and *comEC* were found with the highest expression levels when *H. parasuis* SC1401 were grown on TSA++. *pilA*, has been predicted to encode a type IV pilin, and *pilB* is a traffic nucleotide triphosphatase (NTPase), which could energize assembly of the pilus filament. Both are required for natural competence in certain bacteria (Wang et al., 2003; Meibom et al., 2005). Type IV pilus tends be expressed when nutrition is limited or culture on a solid medium (may be conditionally inducible). TEM and SEM examination of *H. parasuis* grown in TSB++ show no evidence of pilus formation. ComEA, a DNA receptor protein that is reported to be transcriptionally activated by QstR, whose production relies on the quorum-sensing regulator HapR in *V. cholerae* (Provvedi and Dubnau, 1999; Johnston et al., 2014). We hypothesize that the elevated expression level of ComEA may correlate with high cell-density of plate-cultured method in *H. parasuis*. By comparison, the expression levels of three main competence regulons *tfox*/*crp*/*cyaA* are not up-regulated as expected, which indicates that the late-competence genes are not only regulated by these regulatory genes, but also by environmental cues or some other unidentified regulatory network in *H. parasuis*. Moreover, there are three genes (*crp, comEC, comQ*) that were not up-regulated in transformable *H. parasuis* strain SC1401 compared to non-transformable *H. parasuis* strain SH0165. For example, unlike TfoX/Sxy, CRP is a global regulator of carbon and energy metabolism. It activates a broad array of genes related by their roles in obtaining or utilizing alternative carbon or energy sources, or in sparing the wasteful use of the preferred sources (Wu et al., 2015). No similar CRP-S was found in the promoter region of this gene, indicating that, in SH0165, *crp* gene may have a different function other than competence-induction/regulation observed in SC1401.

Natural competence in *H. parasuis* allows us to create allele replacement and study the results of capturing exogenous sibling DNA, thus looking into the mechanism of gene function, the spread of antibiotic-resistance cassettes, the distribution of toxin-encoding phages and the transfer of pathogenicity islands. The phenotype of bacteria is related to the use of DNA for repair, food and/or evolution (Seitz and Blokesch, 2013). However, the high degree of competence diversities in this species pose some questions: Why is competence not universal across all strains, and why among those that are competent are the rates so variable? What are the underlying cues involved in triggering competence besides growth condition and cell density? Further studies could ultimately lead to an improved understanding of molecular/population evolution, competence inducing factors and the fitness of naturally competent cell lines.

## Author contributions

KD, LH, XW, and Y-FC: designed the experiments; SC, XHu, RW, and QZ: performed the experiments with assistance from XHa, QY, YH, and XM. KD, YW, Y-FC, and LH: analyzed the data and wrote the paper. All authors read, commented on and approved the final manuscript.

### Conflict of interest statement

The authors declare that the research was conducted in the absence of any commercial or financial relationships that could be construed as a potential conflict of interest.

## References

[B1] Bailey T. L. Elkan C. (1994). Fitting a mixture model by expectation maximization to discover motifs in biopolymers. Proc. Int. Conf. Intell. Syst. Mol. Biol. 2, 28–36. 7584402

[B2] Beauchamp J. M. Leveque R. M. Dawid S. DiRita V. J. (2017). Methylation-dependent DNA discrimination in natural transformation of *Campylobacter jejuni*. Proc. Natl. Acad. Sci. USA. 114:E8053–E8061. 10.1073/pnas.1703331114 28855338 PMC5617262

[B3] Bigas A. Garrido M. E. de Rozas A. M. Badiola I. Barbé J. Llagostera M. (2005). Development of a genetic manipulation system for *Haemophilus parasuis*. Vet. Microbiol. 105, 223–228. 10.1016/j.vetmic.2004.10.015 15708819

[B4] Bossé J. T. Sinha S. Schippers T. Kroll J. S. Redfield R. J. Langford P. R. (2009). Natural competence in strains of *Actinobacillus pleuropneumoniae*. FEMS Microbiol. Lett. 298, 124–130. 10.1111/j.1574-6968.2009.01706.x 19659732

[B5] Cameron A. D. Redfield R. J. (2008). CRP binding and transcription activation at CRP-S sites. J. Mol. Biol. 383, 313–323. 10.1016/j.jmb.2008.08.027 18761017

[B6] Carlson C. A. Pierson L. S. Rosen J. J. Ingraham J. L. (1983). *Pseudomonas stutzeri* and related species undergo natural transformation. J. Bacteriol. 153, 93–99. 6571730 10.1128/jb.153.1.93-99.1983 PMC217345

[B7] Dai K. Jin J. Wen Y. Wen X. He L. Cao S.. (2016). Complete genome sequence of highly virulent *Haemophilus parasuis* serotype 11 strain SC1401. Genome Announc. 4:e00628–16. 10.1128/genomeA.00628-16 27445368 PMC4956441

[B8] Ding L. Wen X. He L. Yan X. Wen Y. Cao S.. (2016). The arcA gene contributes to the serum resistance and virulence of *Haemophilus parasuis* serovar 13 clinical strain EP3. Vet. Microbiol. 196, 67–71. 10.1016/j.vetmic.2016.10.011 27939158

[B9] Dorer M. S. Fero J. Salama N. R. (2010). DNA damage triggers genetic exchange in *Helicobacter pylori*. PLoS Pathog. 6:e1001026. 10.1371/journal.ppat.1001026 20686662 PMC2912397

[B10] Dorer M. S. Sessler T. H. Salama N. R. (2011). Recombination and DNA repair in *Helicobacter pylori*. Annu. Rev. Microbiol. 65, 329–348. 10.1146/annurev-micro-090110-102931 21682641 PMC3743418

[B11] Fu S. Xu L. Li S. Qiu Y. Liu Y. Wu Z.. (2016). Baicalin suppresses NLRP3 inflammasome and nuclear factor-kappa B (NF-kappaB) signaling during *Haemophilus parasuis* infection. Vet. Res. 47, 80. 10.1186/s13567-016-0359-4 27502767 PMC4977663

[B12] He L. Wen X. Yan X. Ding L. Cao S. Huang X.. (2016). Effect of cheY deletion on growth and colonization in a *Haemophilus parasuis* serovar 13 clinical strain EP3. Gene 577, 96–100. 10.1016/j.gene.2015.11.046 26657038

[B13] Herriott R. M. Meyer E. M. Vogt M. (1970). Defined nongrowth media for stage II development of competence in *Haemophilus influenzae*. J. Bacteriol. 101, 517–524. 5308771 10.1128/jb.101.2.517-524.1970 PMC284936

[B14] Howell K. J. Peters S. E. Wang J. Hernandez-Garcia J. Weinert L. A. Luan S. L.. (2015). Development of a multiplex PCR assay for rapid molecular serotyping of *Haemophilus parasuis*. J. Clin. Microbiol. 53, 3812–3821. 10.1128/JCM.01991-15 26424843 PMC4652097

[B15] Huang J. Wang X. Cao Q. Feng F. Xu X. Cai X. (2016). ClpP participates in stress tolerance and negatively regulates biofilm formation in *Haemophilus parasuis*. Vet. Microbiol. 182, 141–149. 10.1016/j.vetmic.2015.11.020 26711041

[B16] Hülter N. Sørum V. Borch-Pedersen K. Liljegren M. M. Utnes A. L. Primicerio R.. (2017). Costs and benefits of natural transformation in *Acinetobacter baylyi*. BMC Microbiol. 17:34. 10.1186/s12866-017-0953-2 28202049 PMC5312590

[B17] Humbert O. Dorer M. S. Salama N. R. (2011). Characterization of *Helicobacter pylori* factors that control transformation frequency and integration length during inter-strain DNA recombination. Mol. Microbiol. 79, 387–401. 10.1111/j.1365-2958.2010.07456.x 21219459 PMC3075595

[B18] Israel D. A. Lou A. S. Blaser M. J. (2000). Characteristics of *Helicobacter pylori* natural transformation. FEMS Microbiol. Lett. 186, 275–280. 10.1111/j.1574-6968.2000.tb09117.x 10802184

[B19] Jobling M. G. Holmes R. K. (1997). Characterization of hapR, a positive regulator of the *Vibrio cholerae* HA/protease gene hap, and its identification as a functional homologue of the *Vibrio harveyi* luxR gene. Mol. Microbiol. 26, 1023–1034. 10.1046/j.1365-2958.1997.6402011.x 9426139

[B20] Johnsborg O. Eldholm V. Håvarstein L. S. (2007). Natural genetic transformation: prevalence, mechanisms and function. Res. Microbiol. 158, 767–778. 10.1016/j.resmic.2007.09.004 17997281

[B21] Johnston C. Martin B. Fichant G. Polard P. Claverys J. P. (2014). Bacterial transformation: distribution, shared mechanisms and divergent control. Nat. Rev. Microbiol. 12, 181–196. 10.1038/nrmicro3199 24509783

[B22] Jungo F. Bougueleret L. Xenarios I. Poux S. (2012). The UniProtKB/Swiss-Prot Tox-Prot program: a central hub of integrated venom protein data. Toxicon 60, 551–557. 10.1016/j.toxicon.2012.03.010 22465017 PMC3393831

[B23] Juni E. (1978). Genetics and physiology of Acinetobacter. Annu. Rev. Microbiol. 32, 349–371. 10.1146/annurev.mi.32.100178.002025 360969

[B24] Juni E. Janik A. (1969). Transformation of Acinetobacter calco-aceticus (Bacterium anitratum). J. Bacteriol. 98, 281–288. 5781579 10.1128/jb.98.1.281-288.1969 PMC249934

[B25] Kanehisa M. (1997). A database for post-genome analysis. Trends Genet. 13, 375–376. 10.1016/S0168-9525(97)01223-7 9287494

[B26] Kelley L. A. Mezulis S. Yates C. M. Wass M. N. Sternberg M. J. (2015). The Phyre2 web portal for protein modeling, prediction and analysis. Nat. Protoc. 10, 845–858. 10.1038/nprot.2015.053 25950237 PMC5298202

[B27] Krüger N. J. Stingl K. (2011). Two steps away from novelty–principles of bacterial DNA uptake. Mol. Microbiol. 80, 860–867. 10.1111/j.1365-2958.2011.07647.x 21435041

[B28] Li J. Yuan X. Xu L. Kang L. Jiang J. Wang Y. (2016). Efficient construction of *Haemophilus parasuis* mutants based on natural transformation. Can. J. Vet. Res. 80, 281–286. 27733782 PMC5052879

[B29] Li Y. Cao S. Zhang L. Lau G. W. Wen Y. Wu R.. (2016). A TolC-like protein of *Actinobacillus pleuropneumoniae* is involved in antibiotic resistance and biofilm formation. Front. Microbiol. 7:1618. 10.3389/fmicb.2016.01618 27822201 PMC5075564

[B30] Liu M. Zhang L. Huang L. Biville F. Zhu D. Wang M.. (2017). Use of natural transformation to establish an easy knockout method in *Riemerella anatipestifer*. Appl. Environ. Microbiol. 83:e00127–17. 10.1128/AEM.00127-17 28258143 PMC5394337

[B31] Lorenz M. G. Wackernagel W. (1991). High Frequency of Natural genetic transformation of *Pseudomonas stutzeri* in soil extract supplemented with a carbon/energy and phosphorus source. Appl. Environ. Microbiol. 57, 1246–1251. 16348463 10.1128/aem.57.4.1246-1251.1991 PMC182876

[B32] Lorenz M. G. Wackernagel W. (1994). Bacterial gene transfer by natural genetic transformation in the environment. Microbiol. Rev. 58, 563–602. 7968924 10.1128/mr.58.3.563-602.1994 PMC372978

[B33] MacFadyen L. P. (2000). Regulation of competence development in *Haemophilus influenzae*. J. Theor. Biol. 207, 349–359. 10.1006/jtbi.2000.2179 11082305

[B34] Macfadyen L. P. Ma C. Redfield R. J. (1998). A 3',5' cyclic AMP (cAMP) phosphodiesterase modulates cAMP levels and optimizes competence in *Haemophilus influenzae* Rd. J. Bacteriol. 180, 4401–4405. 9721275 10.1128/jb.180.17.4401-4405.1998 PMC107447

[B35] Maughan H. Redfield R. J. (2009a). Extensive variation in natural competence in *Haemophilus influenzae*. Evolution 63, 1852–1866. 10.1111/j.1558-5646.2009.00658.x 19239488

[B36] Maughan H. Redfield R. J. (2009b). Tracing the evolution of competence in *Haemophilus influenzae*. PLoS ONE 4:e5854. 10.1371/journal.pone.0005854 19516897 PMC2689351

[B37] Meibom K. L. Blokesch M. Dolganov N. A. Wu C. Y. Schoolnik G. K. (2005). Chitin induces natural competence in *Vibrio cholerae*. Science 310, 1824–1827. 10.1126/science.1120096 16357262

[B38] Meier P. Wackernagel W. (2003). Mechanisms of homology-facilitated illegitimate recombination for foreign DNA acquisition in transformable *Pseudomonas stutzeri*. Mol. Microbiol. 48, 1107–1118. 10.1046/j.1365-2958.2003.03498.x 12753199

[B39] Mell J. C. Redfield R. J. (2014). Natural competence and the evolution of DNA uptake specificity. J. Bacteriol. 196, 1471–1483. 10.1128/JB.01293-13 24488316 PMC3993363

[B40] Mell J. C. Shumilina S. Hall I. M. Redfield R. J. (2011). Transformation of natural genetic variation into *Haemophilus influenzae* genomes. PLoS Pathog. 7:e1002151. 10.1371/journal.ppat.1002151 21829353 PMC3145789

[B41] Oliveira S. Pijoan C. (2004). *Haemophilus parasuis*: new trends on diagnosis, epidemiology and control. Vet. Microbiol. 99, 1–12. 10.1016/j.vetmic.2003.12.001 15019107

[B42] Palmen R. Buijsman P. Hellingwerf K. J. (1994). Physiological regulation of competence induction for natural transformation in *Acinetobacter calcoaceticus*. Arch. Microbiol. 162, 344–351. 10.1099/00221287-139-2-295 8436948

[B43] Provvedi R. Dubnau D. (1999). ComEA is a DNA receptor for transformation of competent *Bacillus subtilis*. Mol. Microbiol. 31, 271–280. 10.1046/j.1365-2958.1999.01170.x 9987128

[B44] Redfield R. J. Cameron A. D. Qian Q. Hinds J. Ali T. R. Kroll J. S.. (2005). A novel CRP-dependent regulon controls expression of competence genes in *Haemophilus influenzae*. J. Mol. Biol. 347, 735–747. 10.1016/j.jmb.2005.01.012 15769466

[B45] Redfield R. J. Findlay W. A. Bossé J. Kroll J. S. Cameron A. D. Nash J. H. (2006). Evolution of competence and DNA uptake specificity in the Pasteurellaceae. BMC Evol. Biol. 6:82. 10.1186/1471-2148-6-82 17038178 PMC1626085

[B46] Renda B. A. Dasgupta A. Leon D. Barrick J. E. (2015). Genome instability mediates the loss of key traits by *Acinetobacter baylyi* ADP1 during laboratory evolution. J. Bacteriol. 197, 872–881. 10.1128/JB.02263-14 25512307 PMC4325111

[B47] Robert X. Gouet P. (2014). Deciphering key features in protein structures with the new ENDscript server. Nucleic Acids Res. 42, W320–W324. 10.1093/nar/gku316 24753421 PMC4086106

[B48] Seitz P. Blokesch M. (2013). Cues and regulatory pathways involved in natural competence and transformation in pathogenic and environmental Gram-negative bacteria. FEMS Microbiol. Rev. 37, 336–363. 10.1111/j.1574-6976.2012.00353.x 22928673

[B49] Sinha S. Mell J. C. Redfield R. J. (2012). Seventeen Sxy-dependent cyclic AMP receptor protein site-regulated genes are needed for natural transformation in *Haemophilus influenzae*. J. Bacteriol. 194, 5245–5254. 10.1128/JB.00671-12 22821979 PMC3457227

[B50] Solomon J. M. Grossman A. D. (1996). Who's competent and when: regulation of natural genetic competence in bacteria. Trends Genet. 12, 150–155. 10.1016/0168-9525(96)10014-7 8901420

[B51] Sparling P. F. (1966). Genetic transformation of *Neisseria gonorrhoeae* to streptomycin resistance. J. Bacteriol. 92, 1364–1371. 4958881 10.1128/jb.92.5.1364-1371.1966 PMC276432

[B52] Tatusov R. L. Fedorova N. D. Jackson J. D. Jacobs A. R. Kiryutin B. Koonin E. V.. (2003). The COG database: an updated version includes eukaryotes. BMC Bioinformatics 4:41. 10.1186/1471-2105-4-41 12969510 PMC222959

[B53] Wang H. Wu J. H. Ayala J. C. Benitez J. A. Silva A. J. (2011). Interplay among cyclic diguanylate, HapR, and the general stress response regulator (RpoS) in the regulation of *Vibrio cholerae* hemagglutinin/protease. J. Bacteriol. 193, 6529–6538. 10.1128/JB.05166-11 21965573 PMC3232884

[B54] Wang X. Liu W. Zhu D. Yang L. Liu M. Yin S.. (2014). Comparative genomics of *Riemerella anatipestifer* reveals genetic diversity. BMC Genomics 15:479. 10.1186/1471-2164-15-479 24935762 PMC4103989

[B55] Wang Y. Shi W. Chen W. Chen C. (2003). Type IV pilus gene homologs pilABCD are required for natural transformation in *Actinobacillus actinomycetemcomitans*. Gene 312, 249–255. 10.1016/S0378-1119(03)00620-6 12909361

[B56] Waters C. M. Bassler B. L. (2005). Quorum sensing: cell-to-cell communication in bacteria. Annu. Rev. Cell Dev. Biol. 21, 319–346. 10.1146/annurev.cellbio.21.012704.131001 16212498

[B57] Whatmore A. M. Barcus V. A. Dowson C. G. (1999). Genetic diversity of the streptococcal competence (com) gene locus. J. Bacteriol. 181, 3144–3154. 10322016 10.1128/jb.181.10.3144-3154.1999 PMC93770

[B58] Wise E. M. Alexander S. P. Powers M. (1973). Adenosine 3':5'-cyclic monophosphate as a regulator of bacterial transformation. Proc. Natl. Acad. Sci. U.S.A. 70, 471–474. 10.1073/pnas.70.2.471 4346890 PMC433285

[B59] Wroblewski L. E. Peek R. M. Wilson K. T. (2010). *Helicobacter pylori* and gastric cancer: factors that modulate disease risk. Clin. Microbiol. Rev. 23, 713–739. 10.1128/CMR.00011-10 20930071 PMC2952980

[B60] Wu R. Zhao M. Li J. Gao H. Kan B. Liang W. (2015). Direct regulation of the natural competence regulator gene tfoX by cyclic AMP (cAMP) and cAMP receptor protein (CRP) in Vibrios. Sci. Rep. 5:14921. 10.1038/srep14921 26442598 PMC4595672

[B61] Xu Z. Yue M. Zhou R. Jin Q. Fan Y. Bei W.. (2011). Genomic characterization of *Haemophilus parasuis* SH0165, a highly virulent strain of serovar 5 prevalent in China. PLoS ONE 6:e19631. 10.1371/journal.pone.0019631 21611187 PMC3096633

[B62] Yue M. Yang F. Yang J. Bei W. Cai X. (2009). Complete genome sequence of *Haemophilus parasuis* SH0165. J. Bacteriol. 191, 1359–1360. 10.1128/JB.01682-08 19074396 PMC2632009

[B63] Zhang B. Feng S. Xu C. Zhou S. He Y. Zhang L.. (2012). Serum resistance in *Haemophilus parasuis* SC096 strain requires outer membrane protein P2 expression. FEMS Microbiol. Lett. 326, 109–115. 10.1111/j.1574-6968.2011.02433.x 22092746

[B64] Zhang L. Li Y. Dai K. Wen X. Wu R. Huang X.. (2015). Establishment of a successive markerless mutation system in *Haemophilus parasuis* through natural transformation. PLoS ONE 10:e0127393. 10.1371/journal.pone.0127393 25985077 PMC4436007

[B65] Zhang L. Li Y. Dai K. Wen Y. Wen X. Wu R.. (2014). The confirmation of the DNA uptake signal sequence needed for genetic manipulation in *Haemophilus parasuis*. Vet. Microbiol. 173, 395–396. 10.1016/j.vetmic.2014.10.001 25389554

[B66] Zhang L. Li Y. Wen Y. Lau G. W. Huang X. Wu R.. (2016). HtrA is important for stress resistance and virulence in *Haemophilus parasuis*. Infect. Immun. 84, 2209–2219. 10.1128/IAI.00147-16 27217419 PMC4962635

[B67] Zhang P. Hao H. Li J. Ahmad I. Cheng G. Chen D.. (2016). The epidemiologic and pharmacodynamic cutoff values of tilmicosin against *Haemophilus parasuis*. Front. Microbiol. 7, 385. 10.3389/fmicb.2016.00385 27047487 PMC4802331

[B68] Zhao M. Liu X. D. Li X. Y. Chen H. B. Jin H. Zhou R.. (2013). Systems infection biology: a compartmentalized immune network of pig spleen challenged with *Haemophilus parasuis*. BMC Genomics 14:46. 10.1186/1471-2164-14-46 23339624 PMC3610166

[B69] Zhou Q. Feng S. Zhang J. Jia A. Yang K. Xing K.. (2016). Two glycosyltransferase genes of *Haemophilus parasuis* SC096 implicated in lipooligosaccharide biosynthesis, serum resistance, adherence, and invasion. Front. Cell. Infect. Microbiol. 6:100. 10.3389/fcimb.2016.00100 27672622 PMC5018477

