# Supplementary material for: Basic Characterization of Natural Transformation in a Highly Transformable Haemophilus parasuis Strain SC1401
Source: Front Cell Infect Microbiol. 2018 Feb 8;8:32. doi: 10.3389/fcimb.2018.00032 (PMC5809987; doi:10.3389/fcimb.2018.00032)
Supplement: Supplementary file 1 [file Table1.DOCX]

**Table S1. Primers used for quantitative RT-PCR study.**

| **Primers** | **Primer sequences (5 '-3') ^a. b^** |
| --- | --- |
| 16S-F | TGGTAGTCCACGCTGTAAAC |
| 16S-R | AGGATGTCAAGAGTAGGTAAGG |
| *tfox*-SC1401-F | TGTATCCGTACCTTACCTAATATG |
| *tfox*-SC1401-R | CGATTAATTGCTCCATAAAGACG |
| *tfox*-SH0165-F | ATTTAGTAGATAGAGGCGAGATTGA |
| *tfox*-SH0165-R | CAGCAGCATACAAGGCATTATC |
| *cyaA*-SC1401-F | TTTACGCCTTTCATTTCCTGTC |
| *cyaA*-SC1401-R | CACTACCAATCATTCCTTGCTCAG |
| *cyaA*-SH0165-F | TTTACGTCTTTCATTTCCTGTC |
| *cyaA*-SH0165-R | CACTACCAATCATTCCTTGCTCTG |
| *crp*-F | TCAGCCCATTACCATCCATTCAC |
| *crp*-R | AACATCTCCTTGCCTTCTTCATCC |
| *comA*-F | CGAACTCTACTGCCTGCTAC |
| *comA*-R | TTGGTTTGGTGCTATTATTCTGC |
| *comB*-F | TGGAATGGCAAGGCATCAAT |
| *comB*-R | TGGCTTTGTTGTCGCTGTTG |
| *comC*-F | AATGCCACAACATCCATTATATCGT |
| *comC*-R | GTGAGCTGTTTTTGTTGTTGAGC |
| *comD*-F | ATGCTAAGGATAACGCCCAAG |
| *comD*-R | GTCAATCTGAATAATGCCGATAAG |
| *comE*-SC1401-F | AAGACAGTGCCGCCTCAGTT |
| *comE*-SC1401-R | TAATCCGTTGCCTTCTAAATTGC |
| *comE*-SH0165-F | ATCAGCAGTGAACATTTACAACAA |
| *comE*-SH0165-R | TAATCCGTTGCCCTCTAAGTTTC |
| *comF*-F | GCTACTGTGGTGGCTGTGGA |
| *comF*-R | CATTCTGCAAGCGGTGGTT |
| *comEA*-F | AGGCTCAGGCAATTGTGGAA |
| *comEA*-R | AAGTGTCGCTTTCCCGATACC |
| *comEC*-F | AGGCAATCAGTGGCAGTTCA |
| *comEC*-R | TGTCGTTCTTCCGCAATCC |
| *comN*-F | AGTCAGTGATAAACAGATCGCTTG |
| *comN*-R | ACTCTAATCGCCCAGACTTTCC |
| *comP*-F | GCGGAGTTGCCAGAAAGTAGT |
| *comP*-R | AGGTGGAATAAAGTGTGCCATAC |
| *comQ*-F | CAGCAACAGCAAGCCCTACA |
| *comQ*-R | AGTGTATTACTGCGACATTCTACCT |
| *pilA*-SC1401-F | CGAGCAGATAAATCCGATGTCC |
| *pilA*-SC1401-R | GGTCATTGTGTAGCCGTAGC |
| *pilA*-SH0165-F | CGAGCAGATAAATCCGATGTCC |
| *pilA*-SH0165-R | GAGTCATTGTGTAGCCGTAGC |
| *pilB*-F | GCATACCAACGACGCACTCTC |
| *pilB*-R | TCACAGCCTACTCCACGACAC |
| *pilC*-F | AATGCTGGCTTCACATATTCG |
| *pilC*-R | CAATCATCTGTAACATATCCGTAGG |
| *pilD*-F | ATCGCCTTTGTTTACCCTTGAA |
| *pilD*-R | CCCACAAAGTCGAAAGGCTA |
| *pilF*-F | CATTATGGCTTGTTGCTTGTG |
| *pilF*-R | CAGAGTGCGGTAGGTAGTC |
| *comM*-SC1401-F | GCATTATCAAGGAACGCACAATCG |
| *comM*-SC1401-R | GGCAGTAACGGCACTTCAATCG |
| *comM*-SH0165-F | CCGACAGGGCATTATCAAGG |
| *comM*-SH0165-R | CGCACCTTGTGGCAGTAGC |
| *dprA*-F | CGGATTATCGCTCGGAACATTG |
| *dprA*-R | ATGGCACCCTTCGCTATATGG |
| *recA*-SC1401-F | TCAGGTGCTTGGTTCTCTTACG |
| *recA*-SC1401-R | TTCATCATTGCTGTCGCTTGC |
| *recA*-SH0165-F | TCAGGGGCGTGGTTCTCTTAT |
| *recA*-SH0165-R | TCGTCGATACTGTCGCTTGC |
| *ssb*-SC1401-F | GTTAGACAGCCGTAGCAGTGG |
| *ssb*-SC1401-R | TAGCCGCCTTGATTGTAGTTGG |
| *ssb*-SH0165-F | ATCAAGGTGGTGGCTGGAAC |
| *ssb*-SH0165-R | TGGTTGCGGTCTGGTTGC |
| *A4U84_RS02730*-F | GTTCAACGGCACAGCAAGG |
| *A4U84_RS02730*-R | TCACCCATTCCCATCATCACC |
