# Supplementary material for: Basic Characterization of Natural Transformation in a Highly Transformable Haemophilus parasuis Strain SC1401
Source: Front Cell Infect Microbiol. 2018 Feb 8;8:32. doi: 10.3389/fcimb.2018.00032 (PMC5809987; doi:10.3389/fcimb.2018.00032)
Supplement: Supplementary file 2 [file Table2.DOCX]

**Table S2 Natural transformation frequencies of five naturally competent *H. parasuis* strains.**

| **strains** | **transformation frequency**^a^ | ***p*-value compared to TF of SC1401** | **result of significance testing (compared to SC1401)^b^** |
| --- | --- | --- | --- |
| SC1401 | (1.01±0.12)×10^-4^ |  |  |
| EP3 | (0.82±0.09)×10^-4^ | 0.0628 | not significant |
| XJ3103 | (0.55±0.15)×10^-4^ | 0.0091 | significant |
| GA5140 | (0.295±0.18)×10^-4^ | 0.0006 | significant |
| XJ3105 | (0.105±0.2)×10^-4^ | 0.0002 | significant |

^a^ Transformation frequency (TF) means [kanamycin-resistant (Kanr) colony-forming units (CFU)/ total CFU];

^b^ Test of significance is based on *p*-value at 0.05 level.
