# Supplementary material for: Basic Characterization of Natural Transformation in a Highly Transformable Haemophilus parasuis Strain SC1401
Source: Front Cell Infect Microbiol. 2018 Feb 8;8:32. doi: 10.3389/fcimb.2018.00032 (PMC5809987; doi:10.3389/fcimb.2018.00032)
Supplement: Supplementary file 3 [file Table3.DOCX]

**Table S3 Nucleotides and amino acid sequences of *tfox* in ZJ0906.**

Total amino acid number: 207, MW=24320

Max ORF starts at AA pos 1(may be DNA pos 1) for 136 AA(408 bases), MW=16046

10 20 30 40 50 60

1 ATGAAATACATTGATGCAAAAACACAACATATCCGTAATCTTCTTTACCCAATCATTGGC

1 M K Y I D A K T Q H I R N L L Y P I I G

70 80 90 100 110 120

61 GAAACGAAAGCAAAAACCTATTTTTCCTATTATGGAATAATGAAAGATAAAGCTATGTTT

21 E T K A K T Y F S Y Y G I M K D K A M F

130 140 150 160 170 180

121 GCTTTATACAAAGATGATAAATTCTATCTACACATACCAAATCATTGCTTAGAGGAAAAC

41 A L Y K D D K F Y L H I P N H C L E E N

190 200 210 220 230 240

181 ATAGCTAAAAATTTATCTCTTCTTCTCGACTCTCAGACTGGCATTAATCTAAAATCCTTC

61 I A K N L S L L L D S Q T G I N L K S F

250 260 270 280 290 300

241 TATTTAATCCCTCCCGATCTACTTAATGACTTACAACAAGTTTCACATTGGGTCGTCGAA

81 Y L I P P D L L N D L Q Q V S H W V V E

310 320 330 340 350 360

301 AGTGTGAAAGATATTCTCCATACGAAACAAAATCAGTATATGGAAAAGAAAAAATGTATT

101 S V K D I L H T K Q N Q Y M E K K K C I

370 380 390 400 410 420

361 CGGACGCTGCCGAATATGACAATACAACTCGAAAGGACATTAAAAAACTAGGTGTCTACT

121 R T L P N M T I Q L E R T L K N ***** V S T

430 440 450 460 470 480

421 CTATTGATGATTTAGTAGATAGGGGCGAGATTGATATTTTCGTCAACCTCCTTAAAATAG

141 L L M I *** *** I G A R L I F S S T S L K *****

490 500 510 520 530 540

481 GTGTTGATGCTGATCAAGCTTTATTATTTCGACTTCATGGAGCAATAAATCGACAATATA

161 V L M L I K L Y Y F D F M E Q ***** I D N I

550 560 570 580 590 600

541 TCTATACGATTTCAGACAAAACAAAGCAGAATTTACTCAATGATGCAGATAATGCCTTGT

181 S I R F Q T K Q S R I Y S M M Q I M P C

610 620 630

601 ATGCTGCCGGACTCCGAAAGCGATTTAATACTAGATAA

201 M L P D S E S D L I L D
